# An emerging field: An evaluation of biomedical graduate student and postdoctoral education and training research across seven decades

**DOI:** 10.1371/journal.pone.0282262

**Published:** 2023-07-25

**Authors:** Audra Van Wart, Dušanka Djorić, Natalie M. D’Silva, Rebekah Layton, LaKeya Hardy, Elizabeth Suelzer, Julie E. Tetzlaff

**Affiliations:** 1 Division of Biology and Medicine, Brown University, Providence, Rhode Island, United States of America; 2 Department of Microbiology and Immunology, Medical College of Wisconsin, Milwaukee, Wisconsin, United States of America; 3 Vascular Research Laboratory, Providence Veterans Affairs Medical Center, Providence, Rhode Island, United States of America; 4 Office of Graduate Education, University of North Carolina at Chapel Hill, Chapel Hill, NC, United States of America; 5 MCW Libraries, Medical College of Wisconsin, Milwaukee, Wisconsin, United States of America; 6 Division of Pediatric Pathology, Department of Pathology, Medical College of Wisconsin, Milwaukee, Wisconsin, United States of America; Graduate Career Consulting LLC, UNITED STATES

## Abstract

Biomedical graduate student and postdoctoral education and training research has expanded greatly over the last seven decades, leading to increased publications and the emergence of a field. The goal of this study was to analyze this growth by performing a cross-sectional bibliometric analysis using a systematic approach to better understand the publishing trends (including historical vs. emerging themes and research priorities); depth, structure, and evidence-basis of content; and venues for publication. The analysis documented a dramatic increase in biomedical trainee-related publications over time and showed that this area of research is maturing into its own independent field. Results demonstrated that the most frequently published article types in this field are shorter editorial and opinion pieces, and that evidence-based articles are less numerous. However, if current trends continue, projections indicate that by the year 2035, evidence-based articles will be the dominating article type published in this field. Most frequently published topics included career outcomes and workforce characterization and professional development. In recent years, the most cited articles were publications focused on diversity, equity, and inclusion, career outcomes and workforce characterization, and wellness. This study also shows that although a small subset of journals publishes most of this literature, publications are distributed diffusely across a wide range of journals and that surprisingly 68% of these journals have published only a single article on the topic. Further, we noted that the assignment of author- and index-supplied keywords was variable and inconsistent and speculate that this could create challenges to conducting comprehensive literature searches. Recommendations to address this include establishing standard keyword assignment criteria and proposing new index-supplied keywords to improve accessibility of research findings. These changes will be important for bringing visibility of this literature to our community, institutional leaders, national trainee organizations, and funding agencies.

## Introduction

The past seven decades (1950–2019) have seen a steady growth and maturation of biomedical doctoral training programs and postdoctoral training at universities and academic health centers across the country. The number of PhD recipients in the biological and biomedical sciences nearly doubled between 1990 and 2020, with a similar doubling of postdoctoral appointees across these same decades (NSF Survey). In parallel with this growth, professional organizations have emerged with a focus on improving education and training in the biomedical space, such as the Association of American Medical Colleges (AAMC) Group on Research Education and Training (GREAT), and scientific societies representing biomedical subdisciplines (e.g., Society for Neuroscience, American Society for Biochemistry and Molecular Biology, Federation of American Societies for Experimental Biology) have considered education, training, and career development issues of broad importance across biomedical training. Such organizations have helped gather stakeholders and thought leaders to discuss priority issues and best practices and provide both encouragement and a venue for presenting biomedical education research, ideas, and concerns.

Funding agencies have also emphasized innovative approaches for addressing United States training and workforce needs over time, and major funders such as National Institute of Health (NIH), National Science Foundation (NSF), and Howard Hughes Medical Institute (HHMI), have modified their funding mechanisms to promote innovation surrounding the pre/postdoctoral curriculum, mentorship, wellness, and diversity. Examples of funding mechanisms include NIH Broadening Experiences in Scientific Training (BEST), NIH institutional training grants R25/T32, and the NSF Integrative Graduate Education and Research Traineeship. In recent years, calls to action for better career preparation and professional development training, such as Rescuing Biomedical Research (RBR), the Future of Biosciences Graduate and Postdoctoral Training (FOBGAPT I & II conferences) [1], and the NIH Biomedical Workforce Report [2], have led to new funding opportunities to address these concerns. Importantly, funding agencies have placed an increased importance on outcomes assessment and dissemination of this work in peer-reviewed publications, and this has increasingly become an expectation for educators, directors and deans of graduate and postdoctoral training, and for career development professionals that have a growing presence on campuses nationwide. Subsequently, a rich body of literature has accumulated, and biomedical graduate and postdoctoral education has emerged as its own field of research.

The growth of this field follows a similar ontological development as other fields of education research, such as psychology, engineering, nursing, and medicine, which have now developed their own disciplinary identities, literatures, journals, and professional societies. The emergence of evidence-based teaching and learning literatures with disciplinary roots (so-called discipline-based education research or DBER) has been well-studied [3–5], and while the basis of disciplinary divisions can be debated, the chronology and evolution of scientific development of fields within biomedical graduate and postdoctoral education very much parallels these education research subdisciplines. The National Research Council Report [5] acknowledges that professional society statements, field-specific journals, and graduate and postdoctoral roles in these fields are indicators of their maturation into recognized disciplines (see Hendersen et al, 2017 [3] for further discussion, who add the emergence of faculty positions in these fields as an indicator). Further, Fensham [6] argues that markers for emergence of a mature scientific field include academic recognition, professional associations, research conferences, research centers, and research training.

While the biomedical graduate student and postdoctoral education and training research field has most of the hallmarks outlined above, it is missing the type of field-specific journals that can be found in more established educational disciplines. This has presented an obstacle to achieving cohesion of the field and has been the subject of discussion at recent Association of American Medical Colleges Group on Research, Education and Training (AAMC GREAT) annual meetings. For example, it has been challenging to decide in which journals to publish to reach readership of interest, how to locate and stay abreast of the published research, how to determine appropriate reviewers, and ultimately to formally assess research trends across the literature. To address this gap, we performed a bibliometric analysis. This type of analysis is a quantitative methodology to investigate the scientific communication process by measuring and analyzing written documents [7], and allowed us to better define the scope of the literature in the field, report on publication trends, and provide resources and guidance to better connect scholars in the field via improved publication practices. This analysis has allowed us to make important observations and conclusions that could potentially enhance the visibility of this field, resulting in enhanced cultural and institutional awareness, and enrichment of the biomedical trainee experience.

## Methods

### Stage 1: Identify Medical Subject Headings (MeSH) and keywords for the database search

To establish appropriate and comprehensive search terms for this study, a preliminary literature search was conducted with an initial set of search criteria (S1 Table). The initial search pulled only 2,282 results, and did not appear to capture the full range of articles in this subject area. Therefore, the authors [AVW, RL, JT] compiled a list of representative articles from eight general thematic categories related to research conducted on the education and training of biomedical graduate students and postdocs, and these were used to expand the search terms (S2 Table). Categories selected were professional development (PD); career outcomes and workforce (Work); internships and externships (Int); mentoring (Mentor); diversity, equity, and inclusion (DEI); wellness (Well); admissions (Admis); and curriculum (Curr). These categories were generally aligned with the research topic areas covered at AAMC GREAT annual meetings (link). To generate a more comprehensive list of search terms, multiple resources were utilized and included the Yale Medical Subject Heading (MeSH) analyzer (link), which was used as a ‘scoping search’ tool to generate MeSH search terms and keywords; the NIH National Library of Medicine (NLM) MeSH Browser (link) to identify appropriate headings and subheadings; and author-selected keywords from the set of representative articles. The new terms were combined with the original terms from Search 1, to define the final set of search terms used for this study (S1 Fig).

### Stage 2: Database search

The database searches were performed on October 15, 2020, by a medical librarian [ES] who has experience using the advanced search features of each database. Three search engines were used to collect data for this analysis: PubMed**®**, Web of Science™, and Scopus**®**. Search engine characteristics are described in Table 1, and in more detail below. The databases varied in scope and coverage of scientific disciplines, and the type of publications indexed [8, 9]. Searching multiple databases allowed for more complete coverage of published literature, but required the need to deduplicate, cleanup, and standardize the data.

**Table 1:** Overview of database searches.

Platform	Databases Covered	Publisher	No. titles indexed	Scholarly scope	Publications indexed
PubMed	• MEDLINE • PubMed Central	National Library of Medicine	5,200	Biomedical literature	Journal articles
Scopus	• Scopus	Elsevier	25,100*	Wide range of scholarly disciplines	Journal articles, conference abstracts, books
Web of Science Core Collection	• Science citation index • Social Sciences Citation Index • Arts & Humanities Citation index • Conference Proceedings Citation Index • Book Citation Index • Emerging Sources Citation Index • Index Chemicus • Current Chemical Reaction	Clarivate	22,000**	Wide range of scholarly disciplines	Journal articles, conference abstracts, books

* Source: Scopus Content Coverage Guide, updated 2020 (link).

** Source: Web of Science Coverage Details, updated 2021 (link).

#### PubMed®

searches databases from the NLM, including MEDLINE® and PubMed Central®. Articles indexed for MEDLINE® are assigned MeSH terms; a hierarchically organized vocabulary produced by the NLM. Advantages to using PubMed**®** are that it allows results to be exported to a citation manager for ease of analysis, and that it is freely available and widely used and recognized. Some disadvantages of PubMed**®** are that it doesn’t have robust reporting capabilities, it doesn’t search within the full text of an article, and (relevant for the purposes of the present study) it lacks appropriate subject headings and MeSH terms for articles about the education and training of biomedical graduate students and postdocs.

#### Web of Science™

is a citation database that covers all academic disciplines. Some advantages of Web of Science™ are that it has broader coverage of academic literature; the inclusion of more publication types (book chapters, conference proceedings); and that it utilizes standardized sets of subject categories that are assigned to journals making it easier to compare fields. A disadvantage is that it does not incorporate a controlled vocabulary, thus well-built searches rely on strong combinations of keywords. Also, the date coverage of content depends on the subscription to the database.

#### Scopus®

is also a multidisciplinary database that does not employ a controlled vocabulary. It is advantageous for this study because it covers content not indexed by Web of Science™ or PubMed®, and the database is continually adding content from pre-1970.

The PubMed**®** search was designed first and created and run in the legacy version of PubMed**®**. It utilized a combination of MeSH terms and keywords, the keywords were limited to the title and abstract fields, and the search terms were connected with Boolean operators. There were two main components to each search: 1) postgraduate biomedical education, and 2) the eight subject categories selected for this study (S1 Fig). Some terms used to describe (1) postgraduate biomedical education include the MeSH term ‘Education, Medical, Graduate,’ or a combination of doctoral, PhD, or graduate student, and specific biomedical sciences. The two components of the search were combined using the Boolean operator AND. Limit was not set for publication date, but the search was limited to articles published in English.

Each database searched in this study uses its own specialized search syntax. Therefore, the PubMed**®** search was translated for other databases, and when possible, database-specific advanced search techniques were incorporated into the searches.

### Stage 3: Citation management

**Fig 1:**
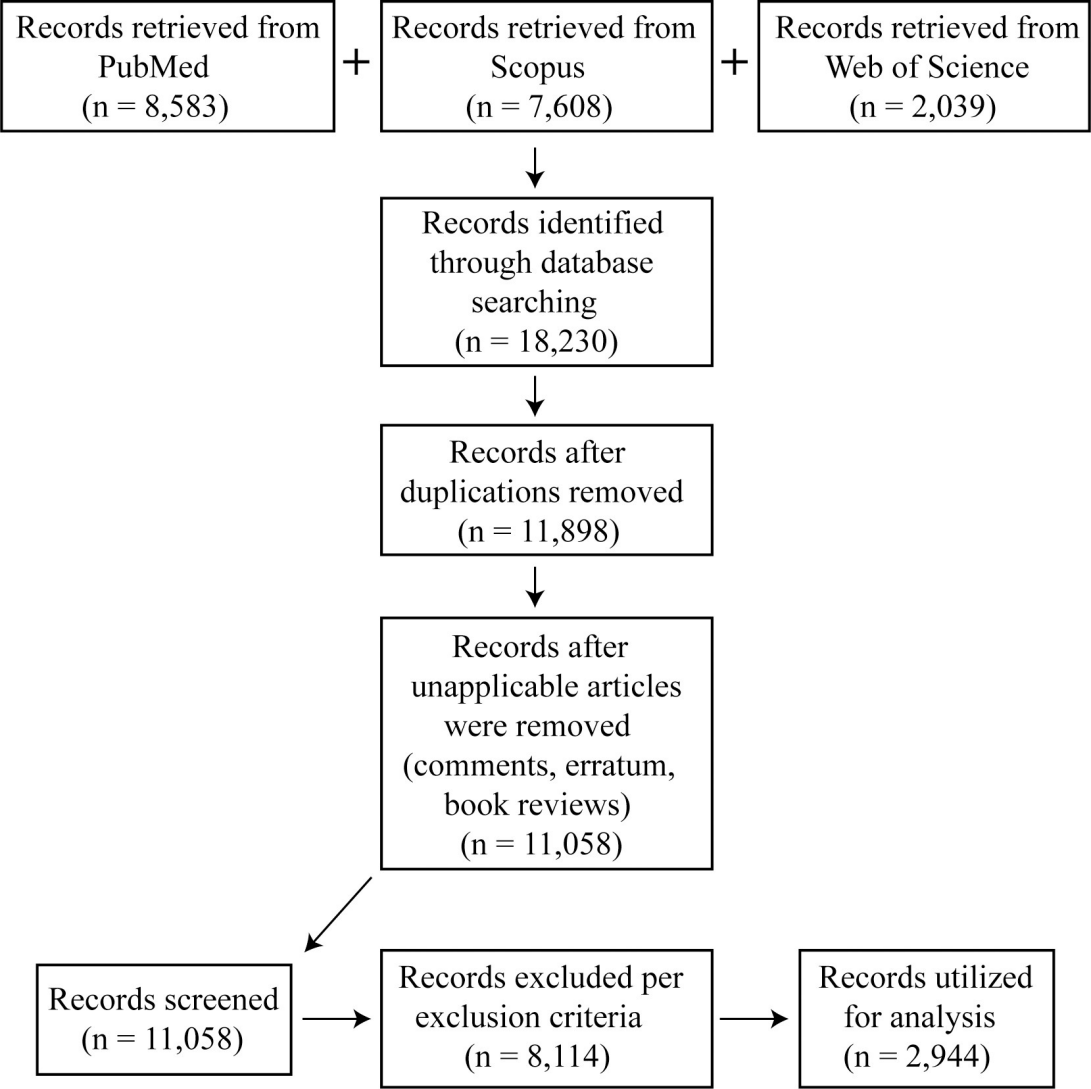
An explanation of the origin of the articles utilized in this study.

The three database searches retrieved 18,230 citations (see Fig 1 for details). Results from the databases were downloaded into an EndNote library. After duplicate articles were removed, a total of 11,898 articles remained for review. The research team determined that items classified as comments, erratum, and book reviews, for example were not applicable to this project. The librarian performed title and publication type searches in EndNote to exclude these types of articles from the blinded screening, and 840 articles were immediately excluded (S3 Table). 11,058 articles remained and were exported from EndNote and imported into Rayyan [10] for screening. Rayyan is a free, web-based resource developed to facilitate blinded screening for systematic reviews or screening projects.

### Stage 4: Selecting and classifying articles for study inclusion

Articles were screened for study inclusion by three teams of two scientists each [ND, JT, RL, AVW, DDJ, LH]. Scientists reviewed each article for inclusion in the study by reading the article title and abstract. If a decision could not be based on title and abstract, the entire article was assessed. Inclusion and exclusion decisions were based on *a priori* defined inclusion and exclusion criteria (S4 Table). Next, scientists assigned each article a category type, which included admissions (Admis), career outcomes and workforce (Work), curriculum (Curr), diversity, equity, and inclusion (DEI), internships and externships (Int), mentoring (Mentor), professional development (PD), and wellness (Well) (S5 Table). If more than one category was appropriate, two were assigned. When two categories were assigned, both were considered equivalent. Scientists assigned each article one research article (RA) type (S6 Table) defined as evidence based (RA1), descriptive (RA2), and editorial (RA3).

Inter-team variability was minimized by thoroughly discussing and agreeing upon criteria for inclusion/exclusion and categorization in advance of the study, and talking through decisions on an initial random sampling of articles. Subsequently, scientists from each team resolved any conflicts through intra-team discussions. If the team could not reach a consensus, or new questions arose about how particular articles fit with the criteria, these were discussed by all scientists at weekly team meetings, and article were resolved by agreement of all scientists. Overall, there were 1,558 intra-team inclusion/exclusion conflicts making up 14% of total articles screened. 8,114 of the screened articles were excluded based on exclusion criteria leaving 2,944 for analysis (stage 5, see Fig 1).

### Stage 5: Analysis

Citation data and inclusion decisions were exported from Rayyan as a CSV file. Excel and SAS 9.4 software were utilized to analyze data and prepare figures and tables. The full set of search results, including the included/excluded articles and category tags, is provided as a supplemental Excel file (S7 Table), and a Bibliography of all included articles is provided as a supplemental RIS file that may be imported into a variety of citation management applications (S1 File).

A biostatistics core (MCW) was consulted for statistical analysis. A descriptive analysis of data from all included articles was performed with a focus on frequencies and percentages of category and article type. For analysis of growth trends of time, a piecewise linear regression was performed for papers from each RA type and for each thematic category tag using R software. Inflection points are reported to indicate the year marking different phases of growth for each type or category (Results and S2 Fig). For prediction of growth trends out to the year 2035 (Table 2 and S3 Fig), the number of articles was modelled using an exponential function: Number of articles = a*exp(b*(year-1995)). The NLIN procedure in SAS software was used to fit nonlinear regression models and parameters were estimated by nonlinear least squares. Nonlinear least-squares estimation involves finding values in the parameter space that minimize the residual of squares. The nonlinear least squares problem was solved using the modified Gauss-Newton method. The estimated parameters a and b are listed in Table 2.

**Table 2:** Projected growth of articles by category and article type. The number of articles published in 2035 was predicted by exponential modeling, and modeling parameters for each category and article type are listed in the table.

**Category**	**Number of articles in 2035**	**Parameter a**	**Parameter b**
PD	585	14.8355	0.0856
DEI	363	3.7931	0.1059
Work	344	13.2515	0.0757
Well	204	1.6933	0.1152
Curr	193	5.1726	0.0807
Mentor	193	1.9936	0.1028
Admis	39	1.3965	0.0741
Int	23	0.2215	0.1108
**Article type**	**Number of articles in 2035**	**Parameter a**	**Parameter b**
RA1	1,026	1.7055	0.1600
RA2	438	8.7967	0.0977
RA3	294	16.8564	0.0715

Additionally, a citation analysis was conducted on a subset of the data to examine citation metrics from RA1 and RA2 articles published between 2014–2021. To include the most current data available, a citation analysis was conducted on October 11, 2021, utilizing data from Web of Science™. A search was performed in Web of Science™ using DOIs for these articles, and data was retrieved for 356 RA1 and 358 RA2 articles, for a total of 714 articles. This analysis excludes RA1 and RA2 articles without DOIs, and articles not indexed in Web of Science™ due to the scope and coverage of the database.

Finally, a keyword analysis was completed using VOSviewer software freely available online (link) [11, 12]. VOSviewer is a tool for constructing and visualizing bibliometric networks and offers text mining functionality that can be used to construct and visualize co-occurrence networks of important terms extracted from a body of scientific literature. In this study, network maps were created to visualize co-occurrence as a weighted parameter of the author-supplied and indexed keywords found in the bibliographic data. The analysis included articles indexed in Scopus®, (2,753 of 2,944). Citation data was exported from Scopus® on December 20, 2021, in a CSV format that included both author keywords and index keywords. According to Scopus®, author keywords are chosen by the author to best reflect the contents of their document. Indexed keywords are chosen by content suppliers and are standardized based on publicly available vocabularies (MeSH and Embase, for example) and are selected by third parties. VOSviewer was used to create two co-occurrence network maps (one for each keyword type) based on bibliographic data. The type of occurrence selected was co-occurrence, the unit of analysis was either author keywords or index keywords, and full counting method was selected. Author-supplied keywords totaled 2,304; of those, 45 met the threshold of 10 minimum occurrences. Index-supplied keywords totaled 4,644 keywords; of those, 82 met the threshold of 100 minimum occurrences.

This article is reported following the Strengthening the Reporting of Observational Studies in Epidemiology (STROBE) reporting guideline [13].

## Results

### Literature distribution and trends

**Fig 2:**
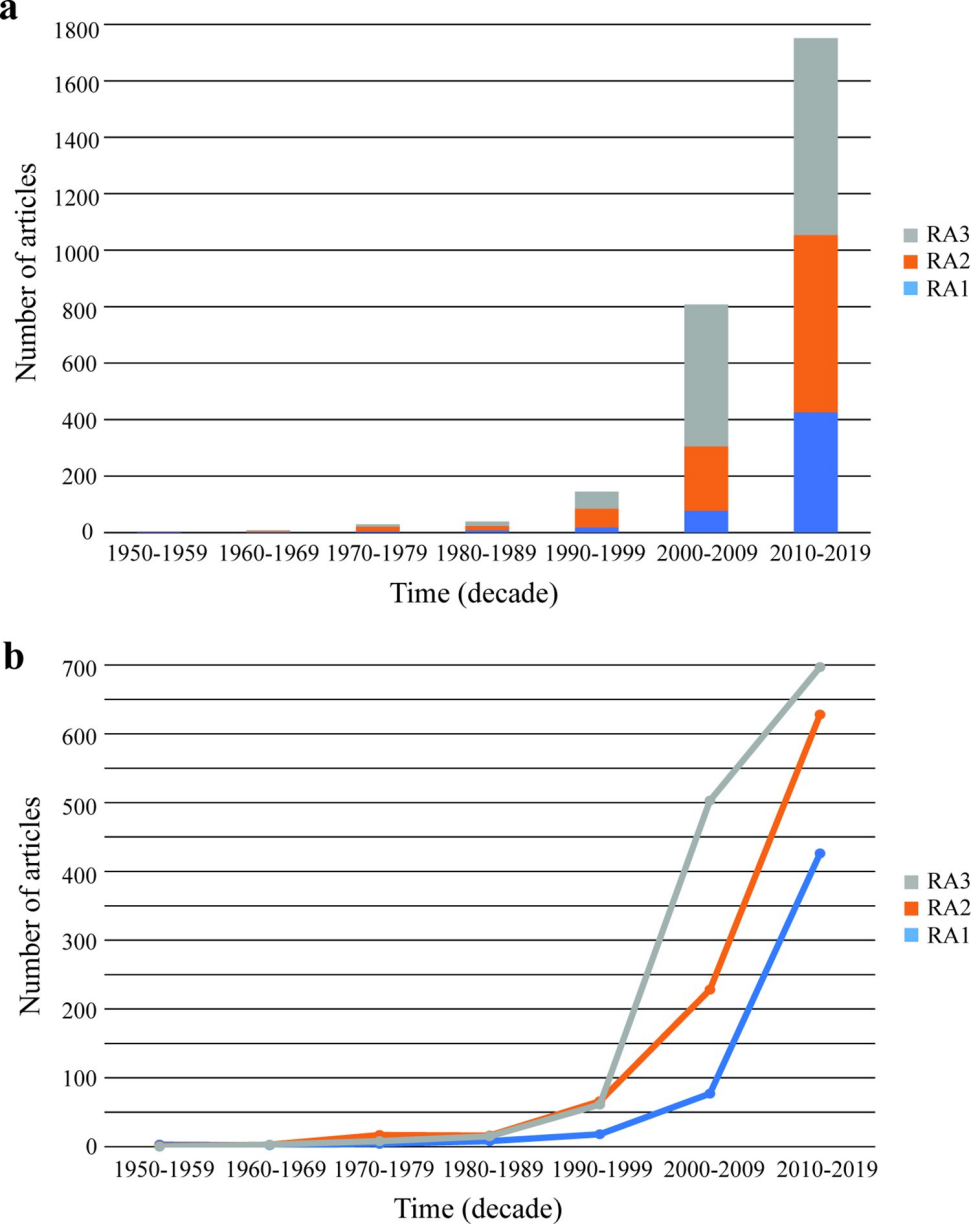
Article types (RA1, RA2, RA3) began to increase at different times during the past 7 decades. This figure includes articles published through 2019.

**Fig 3:**
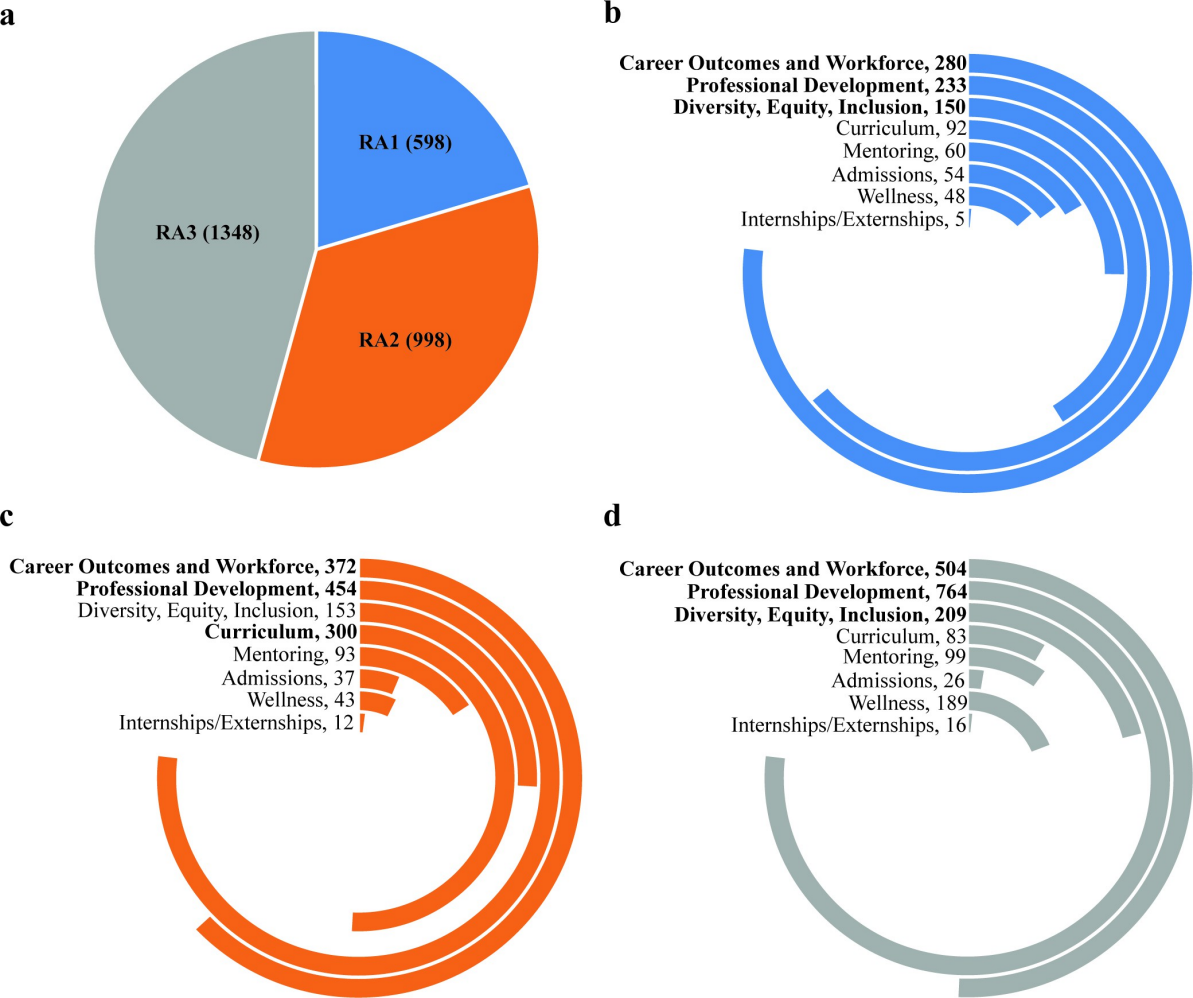
Distribution of article type and category. (a) The pie-chart shows the distribution of included articles across the three research article types, RA1, RA2, and RA3. Within each research article type, RA1 (b), RA2 (c), and RA3 (d), the number of articles for each of the 8 categories were plotted as radial bar charts. It is important to note that some research articles were assigned more than one category, therefore the sum of the numbers for the 8 categories does not equal the number of included research articles.

As a first step toward uncovering trends in the data, the growth in publications over time, as well as distribution of article type and category was assessed. The total number of biomedical graduate student and postdoctoral education and training research related articles has increased over the past seven decades (Fig 2A and 2B). While all three article types have increased over time, they did so at different rates (Fig 2B). Based on piecewise linear regression, RA2 and RA3 articles increased first in 2002 and RA1 articles increased eight years later in 2010 (S2A–S2C Fig). RA1 articles also lagged behind RA2 and RA3 articles based on total articles published. Only 20% of articles published during the period examined were RA1, while 34% and 46% were RA2 and RA3, respectively (Fig 3A).

**Fig 4:**
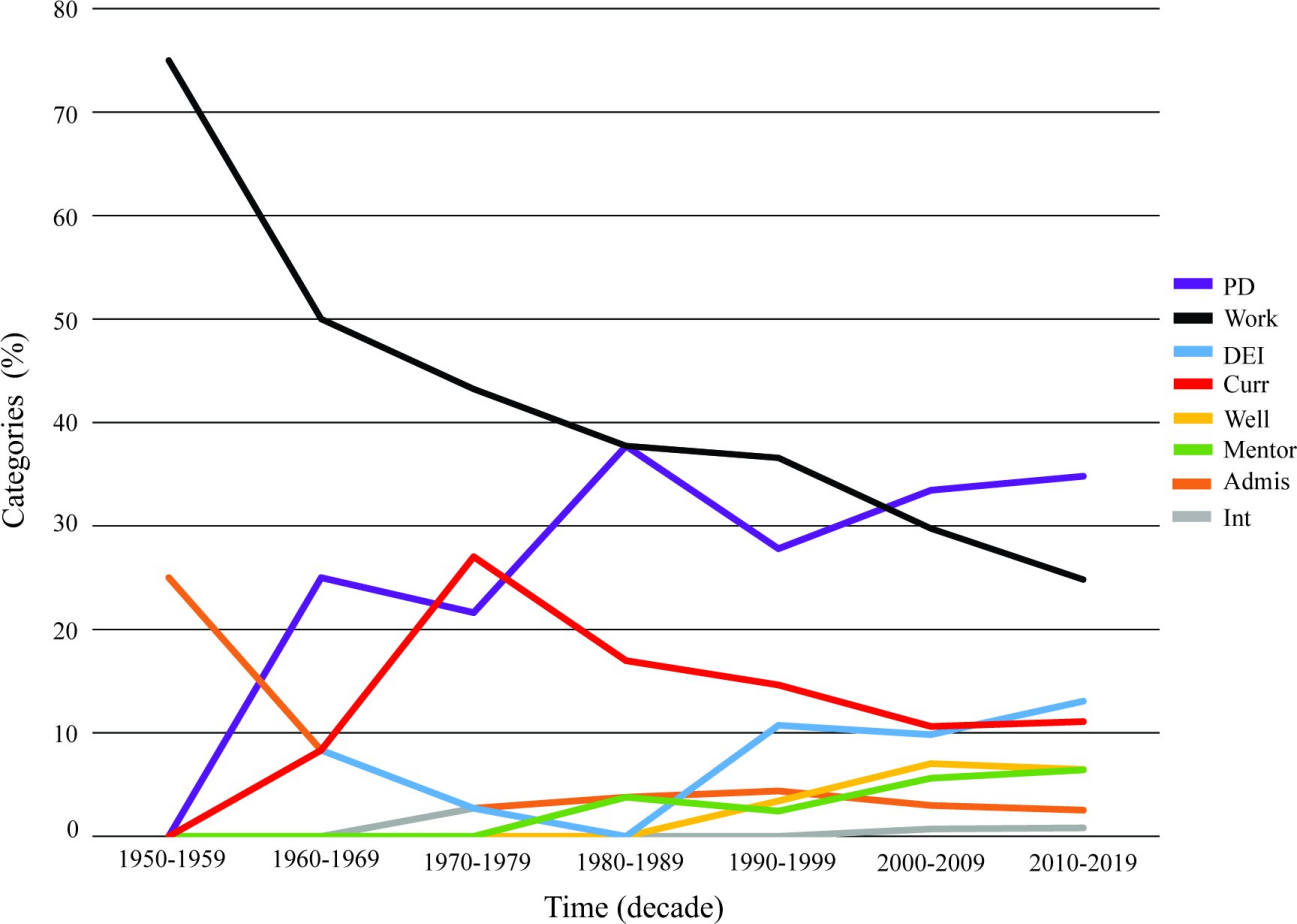
The percentage of total publications represented by each category has changed throughout the past 7 decades. This figure includes articles published through 2019.

The distribution of number of categories assigned varied: 1,612 articles were assigned one category and 1,332 articles were assigned two, for a total of 4,276 category tags. In the case of two category assignment, both categories were equally appropriate. Overall, the categories most frequently published were Work (RA1 = 280; RA2 = 372; RA3 = 504), and PD (RA1 = 233; RA2 = 454; RA3 = 764) (Fig 3B–3D). Conversely, categories involving topics of urgent social need such as Well (RA1 = 48; RA2 = 43; RA3 = 189), Mentor (RA1 = 60; RA2 = 93; RA3 = 99), and DEI (RA1 = 150; RA2 = 153; RA3 = 209), were not published as widely (Fig 3B–3D). Nonetheless, the scope of research has broadened over time, as shown in Fig 4, which demonstrates the emergence of themes as a percentage of total category tags assigned to all papers. Of the category tags assigned to earlier papers, the dominant category was Work. While our data show a growth in the total number of Work-related articles over time, and Work has accounted for the greatest percentage of category tags prior to 2000, the emergence and growth of other thematic areas has cut into the percentage of total articles addressing this topic. The category PD has represented an increasing share of articles over all decades, while DEI (the next most published category) has only recently expanded its share of total articles.

In an assessment of total category tags examined year-by-year across the same time period, each category exhibited a different temporal expression over time with growth inflection points during different eras (S2D–S2K Fig). Based on piecewise linear regression, the category of Work began to increase first in 1995 followed by a second phase of growth across several categories including PD, Admis, Int, Well, and Curr in 1999. During the most recent phase of growth, the category of Mentor articles began to increase in 2000, followed by DEI articles in 2002 (S2D–S2K Fig).

**Fig 5:**
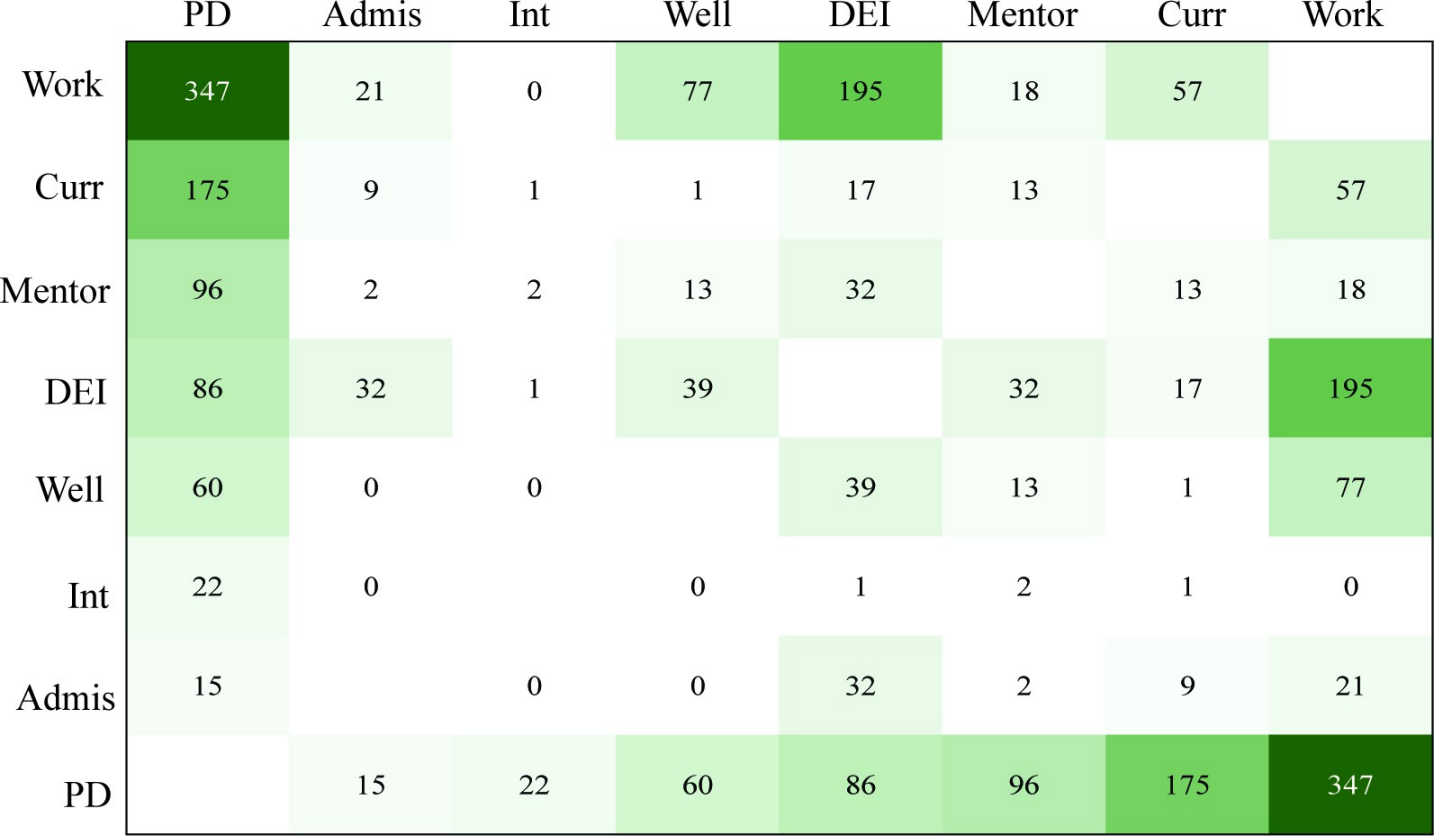
Co-occurring category themes represented by a heatmap showing categories that were published together. The number of articles is represented both as a color scale from light to dark green, as well as numbers on the heatmap, with light green being the lowest number of articles, and dark green indicating the highest number of articles. Only articles with 2 assigned categories were included in this analysis.

To determine which categories were generally published together, we analyzed the 1,332 articles that were assigned the two categories (Fig 5). We found that Work articles were frequently published with PD (347), DEI (195), Well (77), and Curr (57). PD articles frequently occurred with Work (347), Curr (175), Mentor (96), DEI (86), and Well (60). DEI, apart from co-occurring with Work (195) as well as PD (86), was frequently published with other categories such as Well (39), Admis (32), and Mentor (32) demonstrating that there is a wide cross-over between categories published together, consistent with broadening of the field and its research foci.

To assess potential growth of the field, exponential modeling was utilized to predict the number of articles for the year 2035 (an arbitrary year 15 years post current analysis) (Table 2 and S3 Fig). DEI articles are predicted to overcome Work and take place as the second most published category (Table 2). The trend toward increasing rigor of the field by relying upon evidence-based articles is consistent with model-prediction that RA1 articles will be published most numerously in 2035 when compared to RA2 and RA3 articles (Table 2).

### Venues for publications

**Fig 6:**
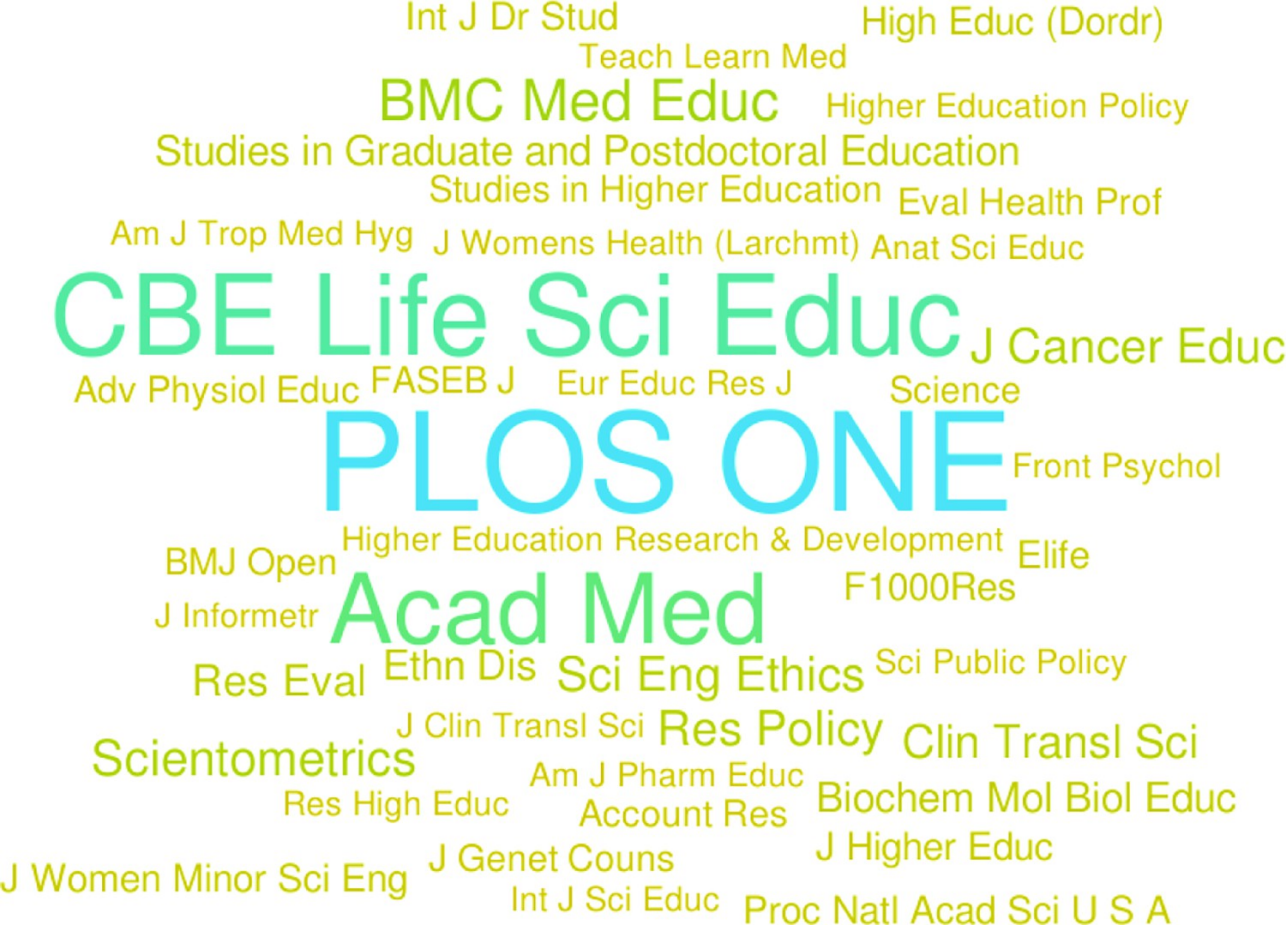
This word cloud (WordItOut) indicates journals that have most frequently published RA1 articles. To accommodate all journal titles on the word cloud, only those journals publishing three articles, or more, were included. Font size directly correlates to the number of articles published. Journal names with the same font color published the same number of articles (link to word cloud).

Analysis revealed 795 unique journals publishing articles related to biomedical graduate student and postdoctoral education and training research. Sixty-eight percent (540) of these journals have published only a single article. Thus, articles in this field are diffusely scattered throughout the literature across many journals, most of which do not normally publish on this topic. Table 3 lists the top ten journals by article type across the seven decades studied as well as the top five of the most recent decade (see * on Table 3). Fig 6 visually represents journals that have published three or more RA1 articles. As indicated in Table 3, PLOS ONE, CBE Life Sciences Education, and Academic Medicine are the top publishers for RA1 articles, publishing 23.7% (142/598) of the RA1 papers identified here. If we look at the top ten journals from each category, we see they have published 36% (214/598) of RA1 articles, 21% (214/998) of RA2 articles, and 65% (884/1348) of RA3 articles. Thus, while the lack of a common journal for this field and the scattered nature of publications may make it difficult to determine in which journal a researcher should search for articles (particularly RA1/2) and publish research on this topic, there is still a subset of journals that publish a notable share of this work.

**Table 3:** Top ten journals by article type across the seven decades studied. Journals marked with a (*) indicate the top five journals of the last decade (2010–2019).

RA1	Number of articles published	RA2	Number of articles published	RA3	Number of articles published
PLOS ONE*	59	Nature*	55	Nature*	640
CBE Life Sci Educ*	45	Acad Med*	36	Science*	135
Acad Med*	38	• Science* • FASEB*	26	EMBO Rep	19
BMC Med Educ*	15	ASEE Annual Conference and Exposition, Conference Proceedings*	23	Physiologist	18
Scientometrics*	12	CBE Life Sci Educ*	19	Elife*	17
J Cancer Educ	11	Sci Eng Ethics	14	Acad Med*	16
• Res Policy • Sci Eng Ethics	10	• Nat Biotechno • Clin Transl Sci • EMBO Rep	12	Mol Biol Cell*	12
Clin Transl Sci	9	• J Cancer Educ • Mol Biol Cell • Elife	11	• Nat Biotechno • Lancet	10
Res Eval	8	• Neuron • AIDS Behav	10	• PLOS Comput Biol • Scientist	9
• Studies in Graduate and Postdoctoral Education • Biochem Mol Biol Educ • Ethn Dis	7	• Anat Sci Educ^§^	8	• Cell • Circ Res • JAMA	8
**Total # of journals**	**258**	**Total # of journals**	**469**	**Total # of journals**	**282**

^§^The book, Postdoc Landscape: The Invisible Scholars was tied for 10^th^ in the RA2 category. This book was removed from the table as books were not considered in this analysis. However, we did want to recognize it as a significant contributor to the literature.

### Citation analysis

Web of Science™ was utilized to tabulate the total number of citations per article (Table 4). The categories with the most citations per article were DEI (18.9), Work (15.7), Well (12.0), and PD (10.0) (Table 4). RA1 and RA2 type articles were cited at similar rates of 13.2 and 10.6 citations per article, respectively (Table 4). Table 5 lists the 10 most frequently cited papers in 2014–2021.

**Table 4:** Citation metrics from RA1 and RA2 articles published between 2014–2021.

	DEI	Work	Well	PD	Mentor	Admis	Curr	Int	RA1	RA2
Total number of citations	3,114	4,767	722	3,111	627	238	909	45	4,712	3,791
Total number of articles	165	303	60	310	78	30	150	10	356	358
Citations per article	18.9	15.7	12.0	10.0	8.0	7.9	6.1	4.5	13.2	10.6

RA3 articles were not included in this analysis.

**Table 5:** The 10 most frequently cited papers in 2014–2021.

Article	Journal	Citations	Category 1	Category 2	Article type
Expectations of brilliance underlie gender distributions across academic disciplines (Leslie, 2015) [32]	Science	464	DEI	Work	RA1
Rescuing US biomedical research from its systemic flaws (Alberts, 2014) [20]	Proc Natl Acad Sci	377	Work	PD	RA2
Gender differences in time spent on parenting and domestic responsibilities by high-achieving young physician-researchers (Jolly, 2014) [33]	Ann Intern Med	307	DEI	None	RA1
Elite male faculty in the life sciences employ fewer women (Sheltzer, 2014) [34]	Proc Natl Acad Sci	154	DEI	PD	RA1
National Institutes of Health addresses the science of diversity (Valantine, 2015) [35]	Proc Natl Acad Sci	149	DEI	Work	RA2
Relationships Among Positive Emotions, Coping, Resilience and Mental Health (Gloria, 2016) [36]	Stress Health	125	Well	None	RA1
Rescuing the physician-scientist workforce: the time for action is now (Milewicz, 2015) [37]	J Clin Invest	102	Work	None	RA2
A generation at risk: young investigators and the future of the biomedical workforce (Daniels, 2015) [38]	Proc Natl Acad Sci	96	Work	PD	RA2
Misconduct Policies, Academic Culture and Career Stage, Not Gender or Pressures to Publish, Affect Scientific Integrity (Fanelli, 2015) [39]	PLOS ONE	96	PD	None	RA1
NIH research funding and early career physician scientists: continuing challenges in the 21st century (Garrison, 2014) [40]	Mol Biol Cell	92	Work	None	RA1

### Keyword analysis

**Fig 7:**
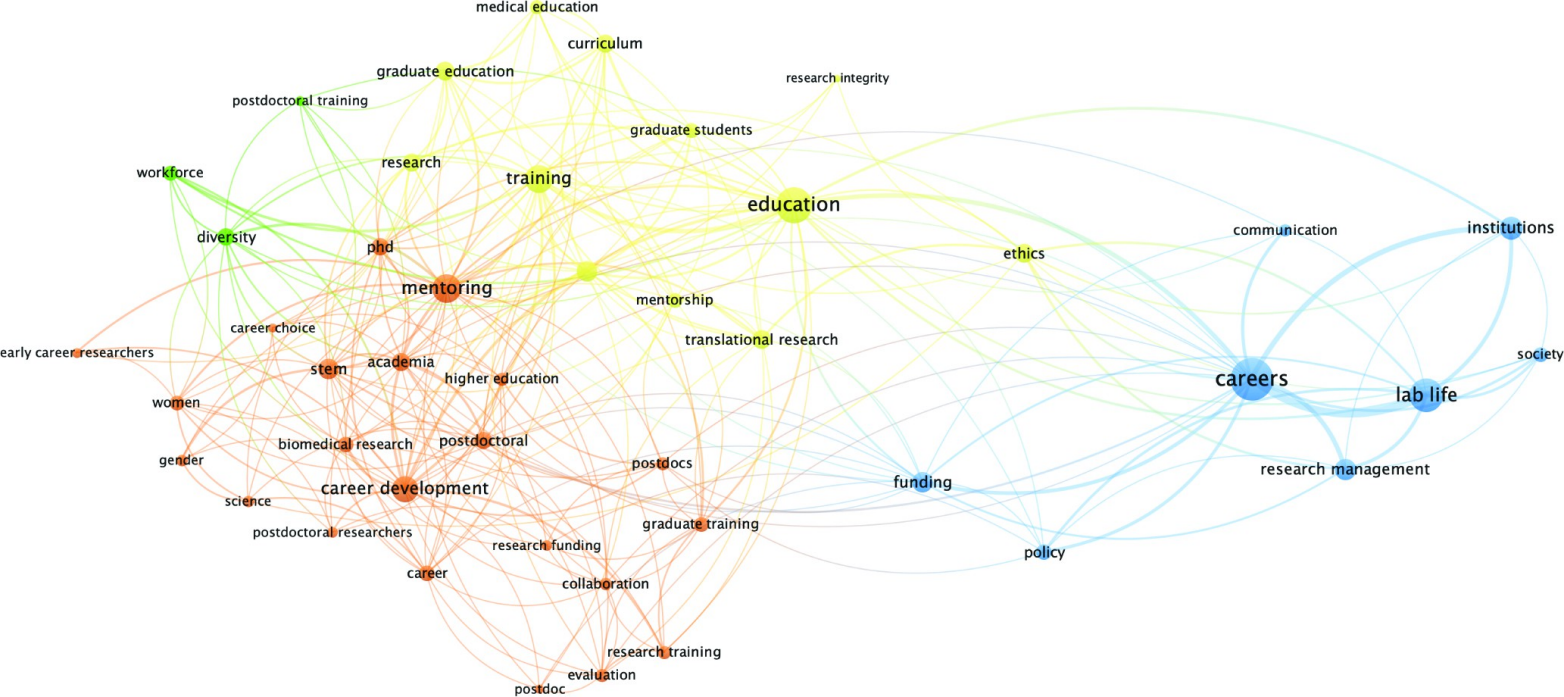
A graphical representation of the most commonly occurring *author*-selected keywords from the SCOPUS^®^ literature search. The size of the circle and word font directly correlates to the frequency of keyword occurrence in the dataset. This map produced 4 clusters of frequently co-occurring keywords (Table 7) and are differentiated by color. Line thickness corresponds to keyword co-occurrence frequency. Mixed line colors indicate the algorithm could not make clear distinctions between the clusters.

**Fig 8:**
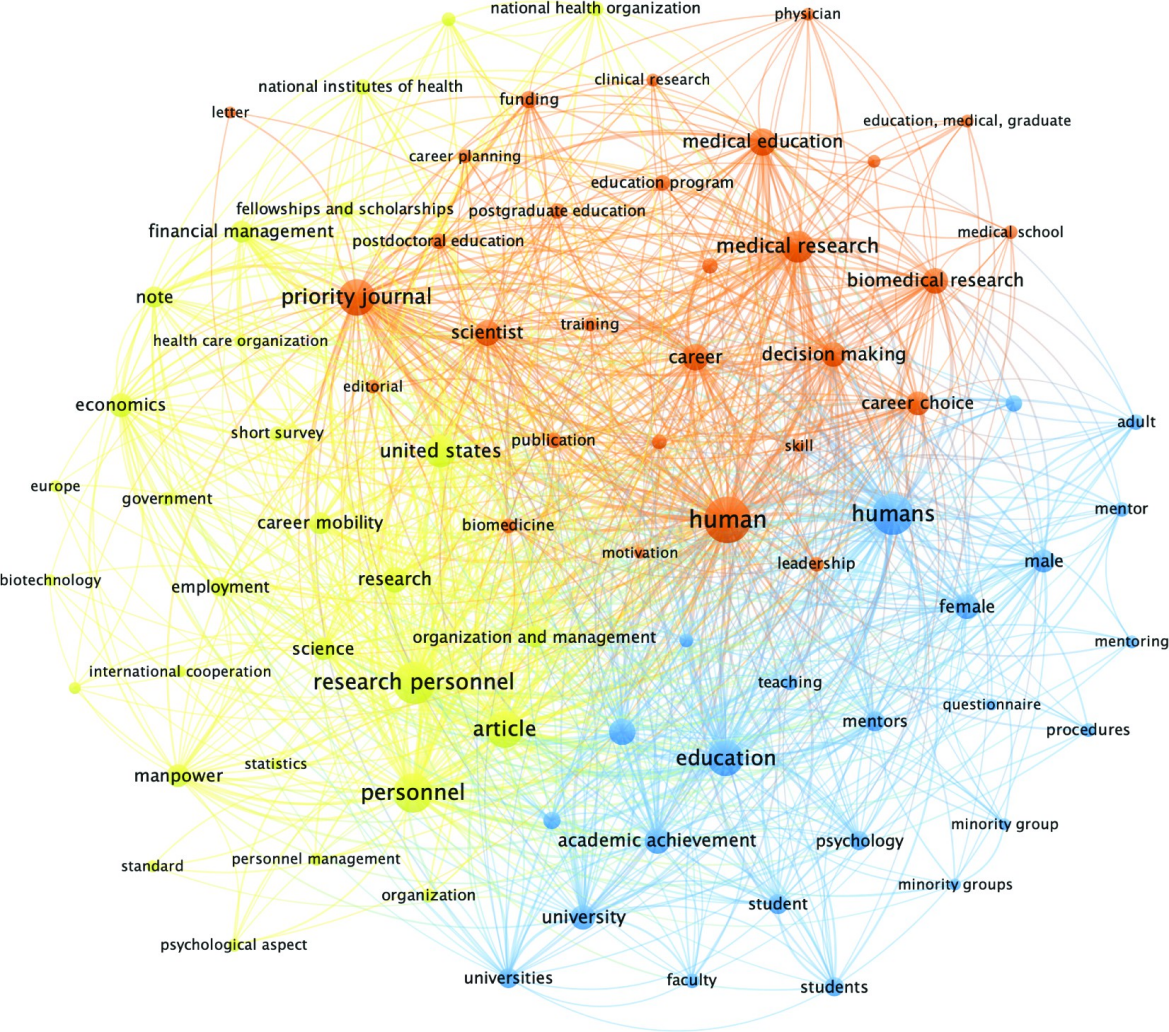
A graphical representation of the most commonly occurring *index*-selected keywords from the SCOPUS^®^ literature search. The size of the circle and word font directly correlates to the frequency of keyword occurrence in the dataset. This map produced 3 clusters of frequently co-occurring keywords (Table 8) and are differentiated by color. Line thickness corresponds to the keywords co-occurrence frequency. Mixed line colors indicate the algorithm could not make clear distinctions between the clusters.

VOSViewer was used to examine article keywords. Four clusters of frequently co-occurring, author-selected keywords (Fig 7 and Table 6) and three clusters of co-occurring, index-selected keywords emerged (Fig 8 and Table 7). Of note are some differences between keyword co-occurrences. Some of the keywords belong to the same cluster but have different patterns of co-occurrence with other keywords (e.g., ’postdocs’ and ‘postdoctoral researchers’ frequently co-occur with ‘mentoring’ while ‘postdoctoral’ does not, yet they refer to the same population, Fig 7 and Table 6). In another example, ‘professional development’ frequently co-occurs with ‘postdoctoral’ and ‘postdoc’, but not ‘postdoctoral researchers’ (Fig 7 and Table 6). Overall, author selected keywords seemed more appropriate and accurately applied (careers, education, mentoring, training) compared to index-selected keywords, which seemed less appropriate (economics, manpower, note) (Table 8). It was also observed that suitable index-selected keywords were underutilized indicating possible indexing errors, or lack of available indexing and/or MeSH terms.

**Table 6:** Four clusters of frequently co-occurring, author-selected keywords identified through VOSViewer.

Cluster 1 (orange)	Cluster 2 (yellow)	Cluster 3 (blue)	Cluster 4 (green)
Academia	Curriculum*	Careers	Diversity*
Biomedical research*	Education*	Communication*	Postdoctoral training
Career	Ethics*	Funding	Workforce*
Career choice*	Graduate education*	Institutions	
Career development	Graduate students	Lab life	
Collaboration	Medical education*	Policy*	
Early career researchers	Mentorship	Research management	
Evaluation*	Professional development	Society*	
Gender*	Research*		
Graduate training	Research integrity		
Higher education	Training		
Mentoring*	Translational research*		
PhD			
Postdoc			
Postdocs			
Postdoctoral researchers			
Research funding			
Research training			
Science*			
STEM			
Women*			

*MeSH term

**Table 7:** Three clusters of co-occurring, index-selected keywords identified through VOSViewer.

Cluster 1 (orange)	Cluster 2 (yellow)	Cluster 3 (blue)
Biomedical research*	Article*	Academic achievement
Biomedicine	Biotechnology	Adult*
Career	Career mobility*	Curriculum*
Career choice*	Economics*	Education*
Career planning	Employment*	Education, graduate*
Decision making*	Europe*	Faculty*
Editorial*	Fellowships and scholarships*	Female*
Education program	Financial management*	Humans*
Education, medical, graduate*	Government*	Male*
Funding	Health care organization	Mentor*
Graduate student	International* cooperation	Mentoring*
Human*	Manpower*	Mentors*
Leadership*	National health organization	Minority group*
Letter*	National Institutes of Health*	Minority groups*
Medical education*	Note	Procedures
Medical research	Organization*	Psychology*
Medical school*	Organization and management	Questionnaire*
Medical student*	Personnel*	Statistics and numerical data*
Motivation*	Personnel management*	Student*
Physician*	Psychological aspect	Students*
Postdoctoral education	Research*	Teacher
Postgraduate education	Research personnel*	Teaching*
Priority journal	Research support as topic*	Universities*
Publication*	Salaries and fringe benefits*	University
Review*	Science*	
Scientist*	Short survey	
Skill	Standard	
Training*	Statistics*	
	United States*	

*MeSH term

**Table 8:** Occurences of author-supplied versus index-supplied keywords.

Author-supplied keyword	Number of occurrences
Careers	94
Education	82
Mentoring	66
Training	49
Lab life	45
Career development	40
Graduate education	30
Research	28
Professional development	28
Diversity	27
STEM	24
Gender	22
Funder	21
Translational research	20
Research management	20
**Index-supplied keyword**	**Number of occurrences**
Research	479
Career choice	414
Economics	397
Manpower	387
Science	387
Male	374
Career mobility	341
Financial management	331
Note	326
Organization and management	309
Mentors	299
Universities	291
Student	284
Employment	267
Students	266

## Discussion

### Growth of a field

In this manuscript, we detail the growth and evolution of the field of biomedical graduate and postdoctoral education and training research through literature analysis. We show a significant increase in total articles over 70 years, with a peak in evidence-based research articles occurring only recently, and a projected growth of 8.4-fold by 2035. The growth of this field, like other fields of education research (e.g., psychology, nursing, engineering) [14, 15] parallels the evolution of structures that place an increasing importance on the execution and publication of research in this area. For example, the sharing and dissemination of assessment data has become increasingly necessary for funding opportunities from federal agencies and non-profits, particularly for past and current mechanisms like the National Institutes of Health NIGMS T32 training grants and R25 mechanisms, National Science Foundation Research Traineeship Program, the Howard Hughes Medical Institute Med into Grad Initiative, and others [16–18]. These funding agencies want to know that biomedical education programs are not only successful but produce generalizable conclusions and/or other resources that can be disseminated and adopted more broadly. Further, faculty and administrators are increasingly using evidence-based approaches to inform programming, curriculum, admissions, and policies, furthering the importance of increasingly sophisticated assessments of programs and processes, and the submission of data for peer review (furthered by organizations such as Understanding Interventions, initiatives like PD|Hub, and funding sources such as NSF/NIH SCISIPBIO biomedical workforce development research grants). Professional societies have, for many years, provided forums for sharing this work in the form of presentations, posters, and workshops (AAMC GREAT, Society for Neuroscience, Graduate Career Consortium, National Postdoctoral Association, and many others), and for the above reasons peer-review and broad dissemination of the work has become increasingly important. Federally funded initiatives in research areas of broad importance across the training pipeline, such as the science of mentorship, have led to new consortia that further develop and promote evidence-based approaches and resources, their adoption, and impact (such as Center for the Improvement of Mentored Experiences in Research, National Research Mentoring Network). Indeed, recent years have also seen a growth in cross-institutional collaborations and consortia to report on national efforts and trends focused on topics from workforce and professional development to climate [19–24]. Altogether these elements have established and shaped a field that will continue to grow and inform the training environment, funding agencies, and policies.

### Emerging priorities in the field

To assess thematic trends over time, we assigned every manuscript included in this study one or two categories from a set of eight categories that cover the range of literature. Our findings indicate that across the decades, publications have heavily focused on the category Work, indicating that this has historically, and continues to be, a foundational issue for the field. This is not surprising given that most of the literature is produced by academic institutions, which focus heavily on training PhDs and postdocs to meet the workforce needs. This is also reflected in the funding priorities across multiple agencies, which aim to support development of a well-trained, competitive, and diverse workforce of biomedical researchers [17, 25]. Over time, the scope of this research has broadened, with PD, DEI, and Curr also representing a high proportion of papers, particularly of evidence-based and descriptive research articles (RA1 and RA2). These emerging themes align with initiatives over the past 15 years to revamp biomedical graduate and training programs to reflect interdisciplinary science [26, 27] and focus on competencies [28], better equip trainees with the skills they need to be successful in a variety of career paths [29, 30], and to better recruit and support a diverse workforce [31]. When we looked at which themes appeared together in papers with multiple categories assigned, we found that papers on Work frequently had a dual theme of PD, DEI or Well, indicating the importance of these three areas for workforce considerations. Other topics frequently covered in conjunction with PD were Curr, Mentor, and DEI.

Interestingly, our citation analysis showed the most cited articles between 2014–2021 were focused on DEI and Work, followed by PD, Well, and Mentor. Thus, despite some of these categories not representing the greatest number of articles to date, they are areas of particular attention and impact in recent years. Based on the trajectory of growth of articles in each of these areas, our data predict that PD and DEI will become the most frequently published categories by 2035.

### Dissemination and accessibility of the work

As with other emerging academic fields, identification of appropriate publication venues for reaching the intended audience has not been straightforward, and the output of this research has outpaced the development of journals focused on this work. Our data show that most publications in this field are distributed across a wide range of journals, most of which publish infrequently in this field. The distributed nature of those articles makes it challenging to find these papers, and reflects the fact that authors are likely relying upon journals in their scientific disciplinary field, despite the potential for broad applicability of their education research across the biomedical sciences disciplines. However, our findings show that there is a small subset of journals that publish the greatest share of the literature in this field, and while these journals do not focus on biomedical education research *per se*, they have evolved to embrace the field (Table 3). Consequently, they have emerged as go-to journals for achieving visibility and are setting the publication standards that will shape how future data are pitched and presented. As the field grows, it is worth considering whether there is space for a dedicated journal for biomedical education research, or room to expand the scope of prominent biomedical journals to include more content on graduate and postdoctoral biomedical education and training.

Given the diffuse nature of the publications in this area, it will be important for authors and readers alike to take steps toward increasing the visibility and accessibility of the work. Our study points to the importance of optimizing search criteria when looking for papers on a given topic, as it is easy to miss the relevant literature. Indeed, we had to expand the search parameters for our initial search to significantly oversample, to capture the range of papers that met our *a priori*-defined inclusion criteria. This included searching multiple databases (Pubmed®, Web of Science^TM^, and Scopus®) to capture publications that may not be indexed by all three, as well as considering which search operations (e.g., author- and index-defined keywords, words in title, MeSH terms) to select when querying databases.

Regarding keywords, we noted that author-provided keywords seemed more appropriate and accurate compared to index-generated keywords indicating that the available selection of appropriate index-generated keywords for this field may be lacking, and new MeSH terms are needed to better represent papers in this field. In some cases, more appropriate MeSH terms for the field were underutilized indicating a lack of awareness or indexing errors. For example, the most appropriate MeSH term currently available for this field ‘education, medical, graduate’ appeared on only 129 articles in this dataset out of a possible 2,944 articles. We suggest utilizing Figs 7 and 8, and Tables 6–8 to select appropriate keywords for publication and conduct literature searches. In addition, authors may consult the many resources available for properly assigning MeSH terms and may contact the National Library of Medicine (NLM) if appropriate MeSH terms have not been applied to their publications.

Based on these findings, we have proposed the addition of the following two MeSH terms to better represent the field: ‘Education, Biological and Biomedical Sciences, Graduate’ and ‘Postdoctoral Training’. We proposed that these terms fall under the heading ‘Education, Professional’, and be listed as a distinct training program to differentiate biomedical education from medical education. While Biological Science Disciplines is an already established MeSH term that describes scientific fields that fall under the scope of biomedical sciences, we feel that the addition of the term “**Biomedical Sciences”** is more recognized and familiar to people within this field and better captures this large, established, and continually evolving field. These terms have been formally submitted to the NLM with the hope they will be added in the next MeSH update. We anticipate that these terms will provide researchers with a more effective way to identify research in this field.

### Study limitations

To perform the literature analysis, we needed to decide on the criteria we would use to define this body of literature. Although our search terms and our inclusion/exclusion criteria targeted biomedical sciences and were aligned with the AAMC GREAT thematic areas for graduate and postdoctoral education research, there is still some level of subjectivity in our definition. Some areas that were excluded could be considered within the realm of biomedical predoctoral and postdoctoral education research, including publications focused on nursing, medicine and residency training, and psychology. However, given that these areas of education research are more developed as fields, with multiple journals dedicated to these audiences, we elected not to include literature from these areas unless the data included or applied to a larger biomedical PhD/postdoc audience. Our data also excluded articles that were not available in English, which limits our ability to assert that these are global publication trends. Our data also do not include articles that are not indexed in the search engines we used, such as preprints and a variety of conference proceedings. While we make predictions on the growth of article types and thematic areas, these predictions cannot anticipate other catalysts for growth in the field. Indeed, the recent COVID-19 pandemic has led to increased concerns of workforce and wellness [21–23], which was not captured in our data. This has also caused institutions to adapt curricula and coursework (remote/hybrid learning), training, and support structures for students/postdocs which will undoubtedly lead to a new line of publications that was previously unanticipated [24, 26]. Similarly, future work is also likely to be shaped by the recent advancement of antiracist movements and increased efforts to develop anti-racist curricula, enhance diversity and inclusivity in the trainee population and workforce, and promote culturally aware mentoring approaches [27–31].

## Conclusions

We have compiled a comprehensive bibliography of publications in the field of biomedical graduate and postdoctoral education and research training based upon our search criteria, from 1951 through October of 2020, along with detailed publishing trends and citation analysis, which should be informative for framing and understanding the growth of the field. Moving forward, this field will continue to be framed by the journal selection and optimization of search terms, and we hope the findings and guidance presented here serve as a useful tool for making these important decisions.

## Supporting information

S1 Table Preliminary search terms. (DOCX)

S2 Table Identification of additional search terms (“S2 Table supplemental fig keyword Selection.xlsx”). (XLSX)

S3 Table EndNote screening, reasons for articles in ‘not applicable’. (DOCX)

S4 Table Inclusion and exclusion criteria. (DOCX)

S5 Table Category types. (DOCX)

S6 Table Research article (RA) types. (DOCX)

S7 Table Full search results with decisions on inclusion and tags (“S7 Table Full_DataSet_2022-04-05.xlsx”). (XLSX)

S1 File RIS file bibliography of all articles that met inclusion criteria (“S1 Bibliography_IncludeArticles_2022-04-05_RIS.txt”). (TXT)

S1 Fig Literature search terms used in the study (Search 2). (DOCX)

S2 Fig The number of publications of each article type (RA1, 2, 3) and in each of the thematic categories, began to increase at different points in time over the decades analyzed in this study. (DOCX)

S3 Fig Prediction of publication rates in 2035. (DOCX)

S1 Data Figure legends and supporting information for excel (S2 and S8) and RIS (S9) files. (DOCX)
