# Supplementary material for: An emerging field: An evaluation of biomedical graduate student and postdoctoral education and training research across seven decades
Source: PLoS One. 2023 Jul 25;18(7):e0282262. doi: 10.1371/journal.pone.0282262 (PMC10368290; doi:10.1371/journal.pone.0282262)
Supplement: S1 Table — (DOCX) [file pone.0282262.s001.docx]

S1 Table: Preliminary Search Terms

A preliminary literature search (Search #1) was conducted utilizing PubMed, Scopus, Web of Science, and ERIC and the search terms listed in the table below. It yielded 2,282 results (after deduplication).

| **PubMed** |  |
| --- | --- |
|  | (“graduate student” OR "graduate students" OR "Education, Medical, Graduate"[Mesh:NoExp] OR postdoc*[TIAB] OR post-doc*[TIAB]) AND (biomedical[TIAB] OR biology[TIAB] OR biochemistry[TIAB] OR biological[TIAB] OR "Biological Science Disciplines"[Mesh] OR "Biochemistry"[Mesh]) AND (Internship* OR externship* OR “experiential learning” OR career OR curriculum OR job OR “Professional development”) |
|  |  |
| **Scopus** |  |
| #1 | TITLE-ABS-KEY(post-doc* OR postdoc* OR (graduate W/3 (student* OR education))) |
| #2 | TITLE-ABS-KEY(biomed* OR biolog* OR biochem*) |
| #3 | TITLE-ABS-KEY(Internship* OR externship* and “experiential learning” OR career OR curriculum OR job OR “Professional development”) |
| #4 | #1 AND #2 AND #3 |
|  |  |
| **Web of Science** |  |
| #1 | TS= ((graduate NEAR/3 (student* OR education)) OR postdoc* OR post-doc*) |
| #2 | TS=(biomed* OR biolog* OR biochem*) |
| #3 | TS=(Internship* OR externship* and “experiential learning” OR career OR curriculum OR job OR “Professional development”) |
| #4 | #1 AND #2 AND #3 |
|  |  |
| **ERIC via Ebsco** |  |
| 1 | DE "Postdoctoral Education" OR DE "Graduate Students" OR postdoc* OR post-doc* OR (graduate W3 (education OR student OR students)) |
| 2 | DE "Biological Sciences" OR DE "Biochemistry" OR DE "Biology" OR DE "Biomedicine" |
| 3 | ((DE "Internship Programs" OR DE "Experiential Learning") OR (DE "Career Development")) OR (DE "Professional Development") OR externship* OR internship* OR career OR curriculum OR job |
| 4 | S1 AND S2 AND S3 |
