# Supplementary material for: An emerging field: An evaluation of biomedical graduate student and postdoctoral education and training research across seven decades
Source: PLoS One. 2023 Jul 25;18(7):e0282262. doi: 10.1371/journal.pone.0282262 (PMC10368290; doi:10.1371/journal.pone.0282262)
Supplement: S3 Table — (DOCX) [file pone.0282262.s003.docx]

# S4 Tables: EndNote screening, reasons for Articles in ‘Not Applicable’. The process for determining unapplicable article included searching the title, notes, type of work and other fields for terms such as interview, viewpoint, erratum, correction, book review, and comment (Table 1). References that were identified in these searches were excluded. Table 2 displays the types and totals of articles that were excluded.

EndNote search strategies to identify initially excluded articles that were not part of the study.

| **EndNote Field** | **Search operator** | **Term** |
| --- | --- | --- |
| Title | contains | **Interview** |
| Title | begins with | **Viewpoint** |
| Title | begins with | **Erratum** |
| Title | begins with | **Correction** |
| Notes | contains | **Biography** |
| Title | contains | **Scientist to watch** |
| Any field | contains | **Book review** |
| Title | contains | **Corrigendum** |
| Title | begins with | **Comment** |
| Title | begins with | **First person** |
| Title | begins with | **Notices** |

Article types immediately excluded from the study.

| **Article type** | **Number** |
| --- | --- |
| Biography | 274 |
| Book review | 8 |
| Comment | 40 |
| Correction | 55 |
| Erratum | 42 |
| Interview | 395 |
| Notice/Announcement | 26 |
| Grand Total | 840 |
