# Supplementary material for: An emerging field: An evaluation of biomedical graduate student and postdoctoral education and training research across seven decades
Source: PLoS One. 2023 Jul 25;18(7):e0282262. doi: 10.1371/journal.pone.0282262 (PMC10368290; doi:10.1371/journal.pone.0282262)
Supplement: S4 Table — (DOCX) [file pone.0282262.s004.docx]

# S5 Table: Inclusion and exclusion criteria

|  | **Included** | **Excluded** |
| --- | --- | --- |
| **Population** | Biomedical   - Graduate students - Postdoctoral fellows - MD/PhD | - Residents - Fellows - Clinicians - General practitioners - Faculty - Research personnel - Medical students - Nursing - Dentistry |
| **Terminal degrees** | - PhD - MD - MD/PhD | - Masters (terminal) |
| **Research focus** | - Biomedical science - Clinical - Translational | - Clinical departmental research (for example, surgery) - Social Science |
