# Supplementary material for: An emerging field: An evaluation of biomedical graduate student and postdoctoral education and training research across seven decades
Source: PLoS One. 2023 Jul 25;18(7):e0282262. doi: 10.1371/journal.pone.0282262 (PMC10368290; doi:10.1371/journal.pone.0282262)
Supplement: S5 Table — (DOCX) [file pone.0282262.s005.docx]

# S6 Table: Category types

| **Category** | **Abbreviation** | **Definition** |
| --- | --- | --- |
| **Admissions** | Admis | - Recruitment (pipeline programs) - Programmatic evaluation - Student selection - Matriculation |
| **Career outcomes and workforce** | Work | - Tracking career outcomes - Work that characterizes the ecosystem and demographics of the biomedical workforce |
| **Curriculum** | Curr | - Design and implementation of coursework, formal education, or training programs. |
| **Diversity, Equity, and Inclusion** | DEI | - Representation, inclusion, training approaches, and/or outcomes of underrepresented groups in graduate and postdoc training - Achieving a diverse biomedical workforce - International trainee experience |
| **Internships and externships** | Int | - Experiential learning in the form of internships, site visits, or job simulation - Development of scientific skills and/or professional skills |
| **Mentoring** | Mentor | - Scientific mentorship - Professional mentorship - Mentor-mentee relationships - Mentor training - Novel mentorship approaches |
| **Professional development** | PD | - Development of skills and competencies - Grant writing - Entrepreneurship - Communication - Self-assessment - Career planning and exploration |
| **Wellness** | Well | - Physical and mental health and well-being of trainees and trainers - Reports on topics such as   - Work/life balance   - Anxiety   - Depression |
