# Supplementary material for: An emerging field: An evaluation of biomedical graduate student and postdoctoral education and training research across seven decades
Source: PLoS One. 2023 Jul 25;18(7):e0282262. doi: 10.1371/journal.pone.0282262 (PMC10368290; doi:10.1371/journal.pone.0282262)
Supplement: S6 Table — (DOCX) [file pone.0282262.s006.docx]

# S7 Table: Research Article (RA) types

| **Abbreviation** | **Definition** |
| --- | --- |
| **RA1** | - Evidence-based - Must have methods and results |
| **RA2** | - Some evidence - Methods not required - Conference reports - Conference proceedings - Program descriptions - Book chapters - Posters with data - Abstracts with data - Fellowship descriptions - Curriculum descriptions |
| **RA3** | - Brief (generally a few paragraphs) - Editorial - Commentary - Opinion |
