# Supplementary material for: An emerging field: An evaluation of biomedical graduate student and postdoctoral education and training research across seven decades
Source: PLoS One. 2023 Jul 25;18(7):e0282262. doi: 10.1371/journal.pone.0282262 (PMC10368290; doi:10.1371/journal.pone.0282262)
Supplement: S1 Fig — (DOCX) [file pone.0282262.s009.docx]

S3 Fig: Literature search terms used in the study (Search #2)

**PubMed search**

(Mentors[Mesh] OR Mentoring[Mesh] OR "Social Support"[Mesh] OR "Stress, Psychological"[Mesh] OR "Mental Health"[Mesh] OR "Social Isolation"[Mesh] OR "Self care"[Mesh] OR "Emotions"[Mesh] OR "Loneliness"[Mesh] OR "Work Life Balance"[Mesh] OR "Work Performance"[Mesh] OR "Cultural Diversity"[Mesh] OR "Ethnic Groups"[Mesh] OR "Career Choice"[Mesh] OR "Minority Groups"[Mesh] OR "Sex Factors"[Mesh] OR "Social Identification"[Mesh] OR "Sex Distribution"[Mesh] OR "Prejudice "[Mesh] OR "Fellowships and Scholarships"[Mesh] OR "College Admission Test"[Mesh] OR "Educational Measurement"[Mesh] OR "Aptitude Tests"[Mesh] OR "School Admission Criteria"[Mesh] OR "Program Development"[Mesh] OR "Career Choice"[Mesh] OR "Workforce"[Mesh] OR "Salaries and fringe benefits"[Mesh] OR "Employment "[Mesh] OR "Career Mobility"[Mesh] OR mentor*[TIAB] OR “social support” [TIAB] OR “psychological stress” [TIAB] OR “burn-out” [TIAB] OR burnout[TIAB] OR “mental health” [TIAB] OR “social isolation” [TIAB] OR “self care” [TIAB] OR emotion*[TIAB] OR lonely[TIAB] OR loneliness[TIAB] OR “work life balance” [TIAB] OR “work performance” [TIAB] OR diversity[TIAB] OR ethnic*[TIAB] OR career*[TIAB] OR “minority group*”[TIAB] OR “sex factors” [TIAB] OR “social isolation” [TIAB] OR “sex distribution” [TIAB] OR prejudice*[TIAB] OR racism[TIAB] OR sexism[TIAB] OR “gender discriminat*”[TIAB] OR fellowship*[TIAB] OR scholarship*[TIAB] OR “college admission test*”[TIAB] OR “educational measurement” [TIAB] OR “educational performance” [TIAB] OR “aptitude test*”[TIAB] OR admission*[TIAB] OR “program development” [TIAB] OR workforce[TIAB] OR salar*[TIAB] OR “fringe benefit*”[TIAB] OR employment[TIAB]) AND ((("Education, Medical, Graduate"[Mesh:NoExp] OR "Education, Graduate"[Mesh:NoExp] OR “graduate school*”[TIAB] OR “graduate student*”[TIAB] OR doctoral*[TIAB] OR PhD[TIAB] OR Ph.D.[TIAB]) AND ("Biomedical Research"[Mesh] OR "Biological Science Disciplines"[Mesh] OR Anatomy[TIAB] OR Biochemistry[TIAB] OR Biology[TIAB] OR Biomedical[TIAB] OR Biophysics[TIAB] OR Neuroscience*[TIAB] OR Pharmacology[TIAB] OR Physiology[TIAB] OR Toxicology[TIAB])) OR postdoc*[TIAB] OR post-doc*[TIAB] OR "Research Personnel"[Mesh]) Limited to English Language.

**Web of Science**

TS=((mentor* OR "social support" OR "psychological stress" OR "burn-out" OR burnout OR "mental health" OR "social isolation" OR "self care" OR emotion* OR lonely OR loneliness OR "work life balance" OR "work performance" OR diversity OR ethnic* OR career* OR "minority group*" OR "sex factors" OR "social isolation" OR "sex distribution" OR prejudice* OR racism OR sexism OR "gender discriminat*" OR fellowship* OR scholarship* OR "college admission test*" OR "educational measurement" OR "educational performance" OR "aptitude test*" OR admission* OR "program development" OR workforce OR salar* OR "fringe benefit*" OR employment ) AND ((("graduate school*" OR "graduate student*" OR doctoral* OR PhD OR Ph.D. ) AND (Anatomy OR Biochemistry OR Biology OR Biomedical OR Biophysics OR Neuroscience* OR Pharmacology OR Physiology OR Toxicology ) ) OR postdoc* OR post-doc* OR "Research Personnel")) Limited to English Language.

**Scopus**

TITLE-ABS-KEY((mentor* OR "social support" OR "psychological stress" OR "burn-out" OR burnout OR "mental health" OR "social isolation" OR "self care" OR emotion* OR lonely OR loneliness OR "work life balance" OR "work performance" OR diversity OR ethnic* OR career* OR "minority group*" OR "sex factors" OR "social isolation" OR "sex distribution" OR prejudice* OR racism OR sexism OR "gender discriminat*" OR fellowship* OR scholarship* OR "college admission test*" OR "educational measurement" OR "educational performance" OR "aptitude test*" OR admission* OR "program development" OR workforce OR salar* OR "fringe benefit*" OR employment ) AND ((("graduate school*" OR "graduate student*" OR doctoral* OR PhD OR Ph.D. ) AND (Anatomy OR Biochemistry OR Biology OR Biomedical OR Biophysics OR Neuroscience* OR Pharmacology OR Physiology OR Toxicology ) ) OR postdoc* OR post-doc* OR "Research Personnel")) Limited to English Language.
