# Supplementary material for: An emerging field: An evaluation of biomedical graduate student and postdoctoral education and training research across seven decades
Source: PLoS One. 2023 Jul 25;18(7):e0282262. doi: 10.1371/journal.pone.0282262 (PMC10368290; doi:10.1371/journal.pone.0282262)

S10 Figure: The number of publications of each article type (RA1, 2, 3) and in each of the thematic categories, began to increase at different points in time over the decades analyzed in this study. The number of articles published annually was plotted for each thematic area and for each article type (RA1, 2, 3). Piecewise linear regression was used to find the cutoff time, as shown in panels A – K.

**A. RA1 cut off year = 2010**


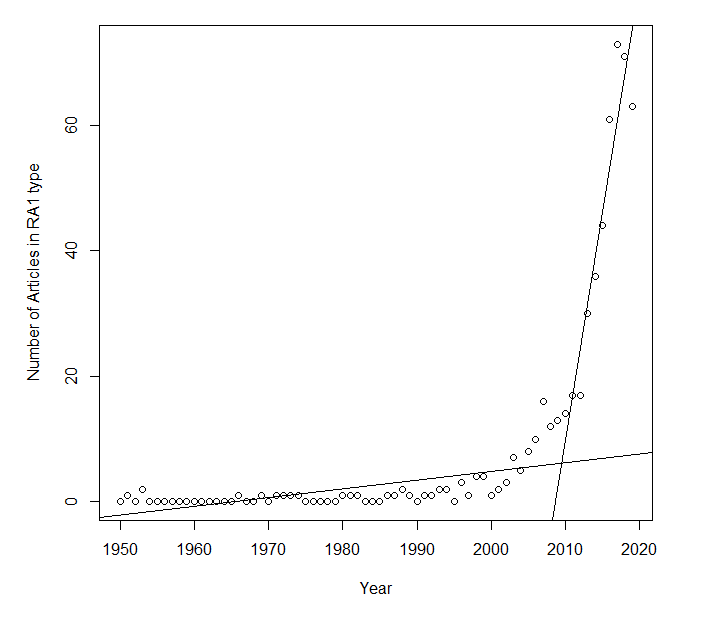


**B. RA2 cut off year = 2002**


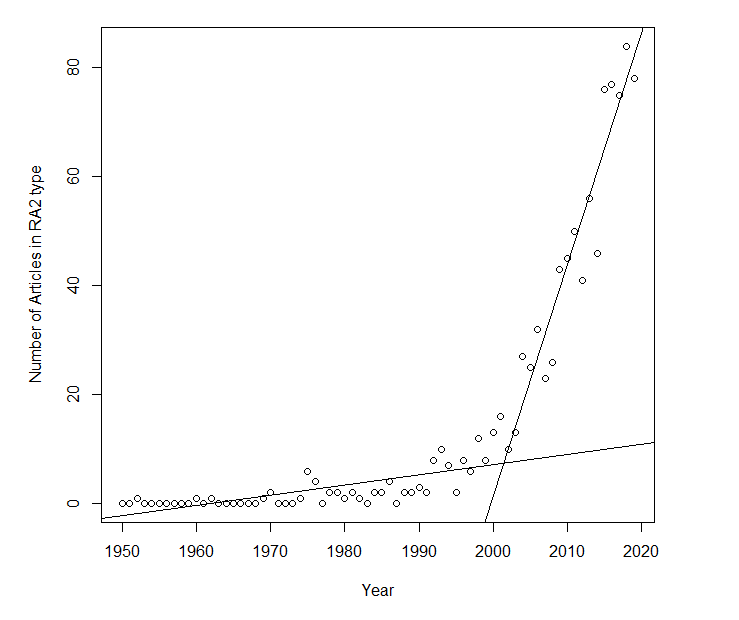


**C. RA3 cut off year = 2002**


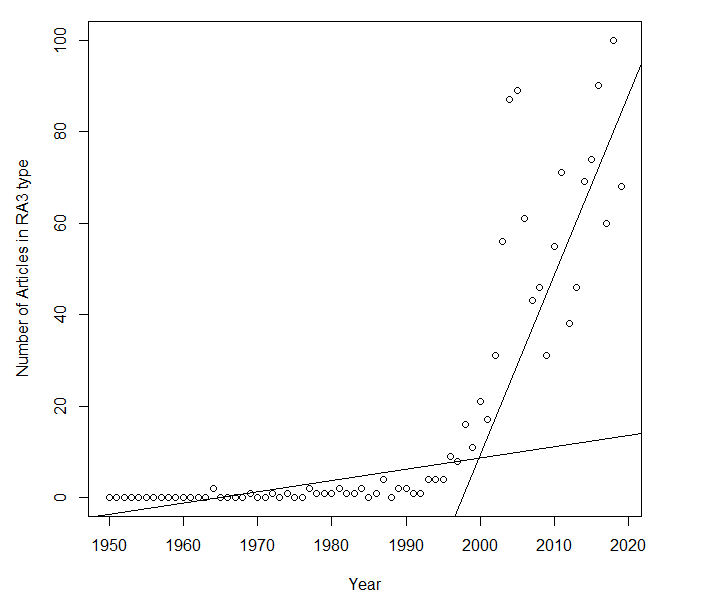


**D. Professional Development cut off year = 1999**


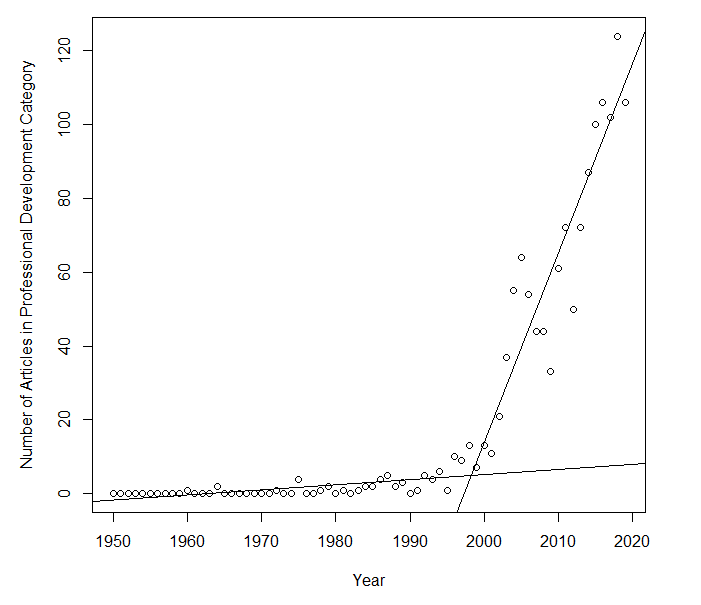


**E. Admissions cut off year = 1999**


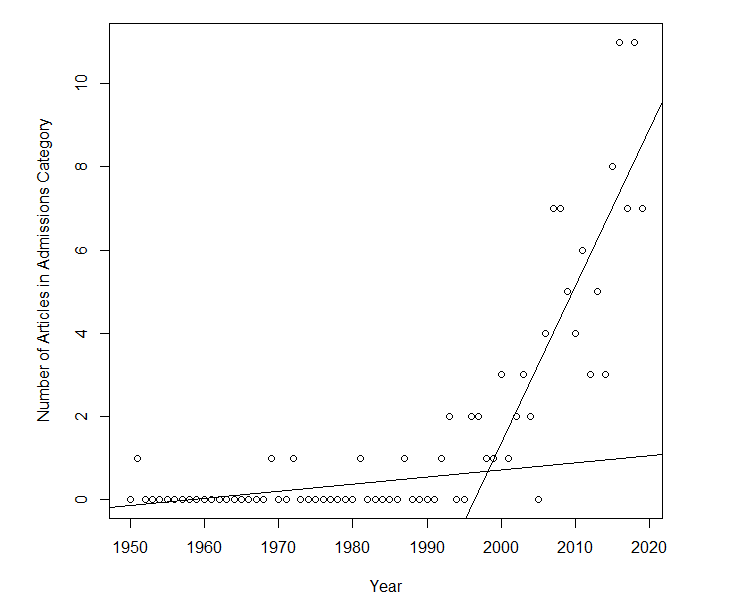


**F. Internship cut off year = 1999**


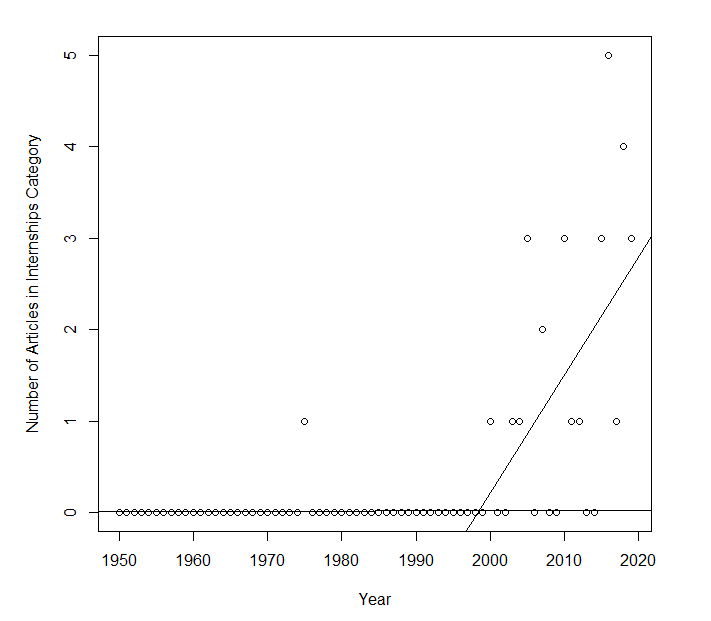


**G. Wellness cut off year = 1999**


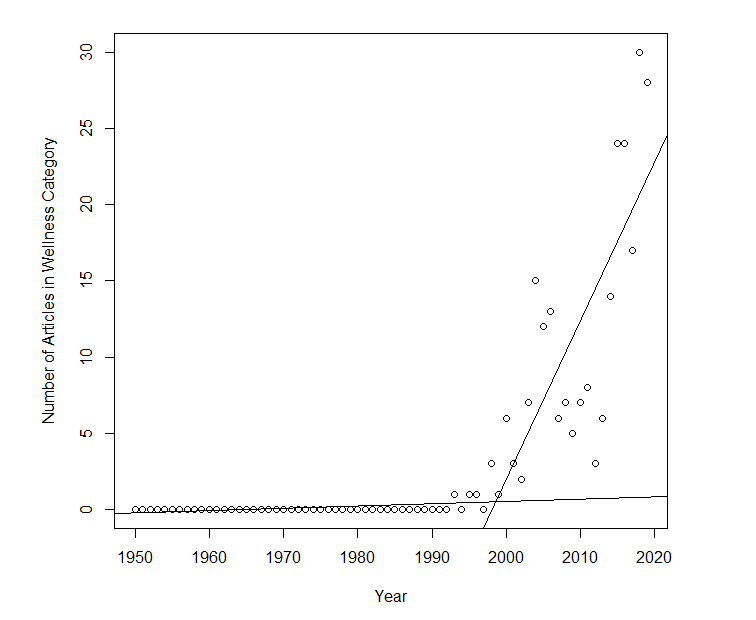


**H. DEI cut off year = 2002**


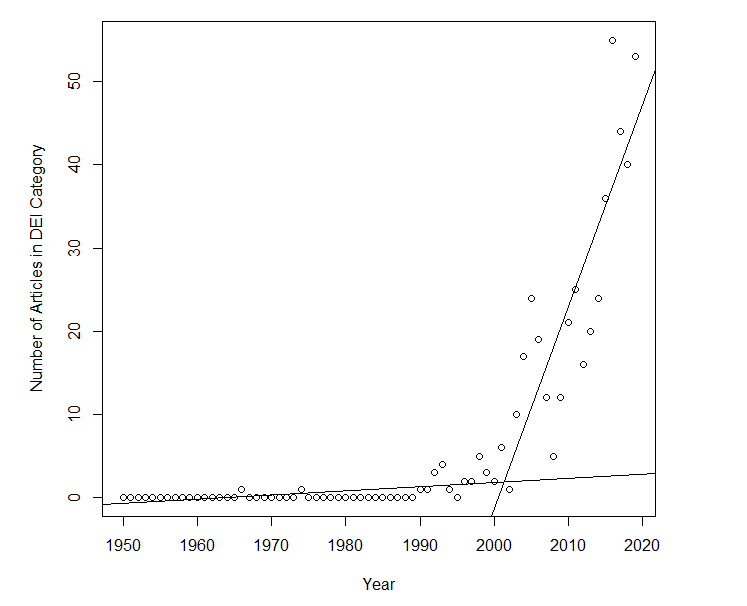


**I. Mentoring cut off year = 2000**


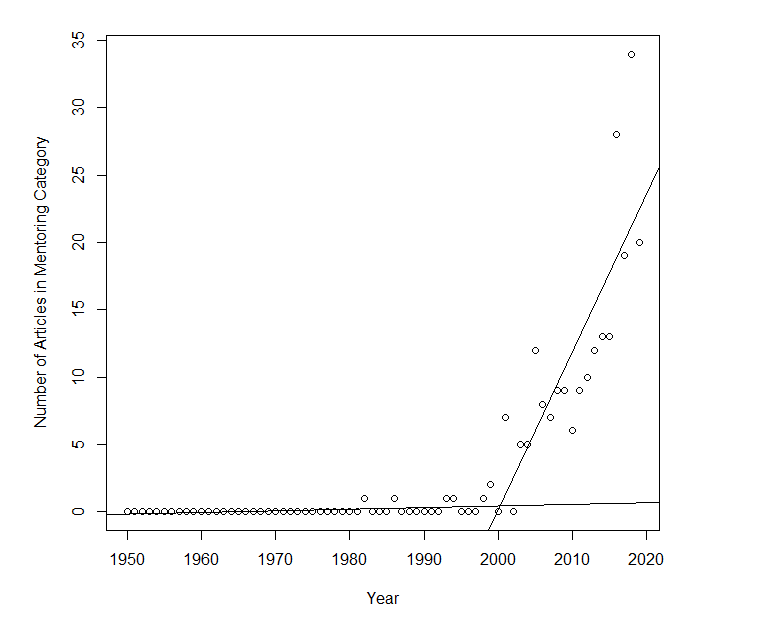


**J. Curriculum cut off year = 1999**


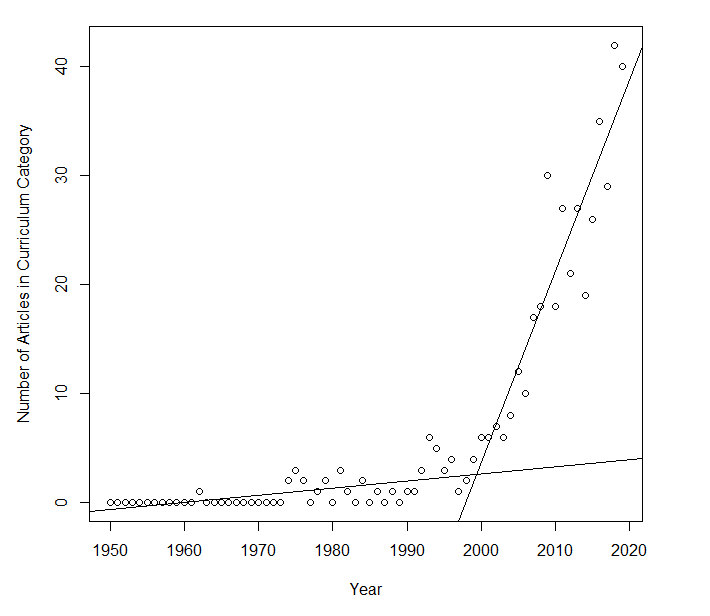


**K. Career Outcomes and Workforce cut off year = 1995**


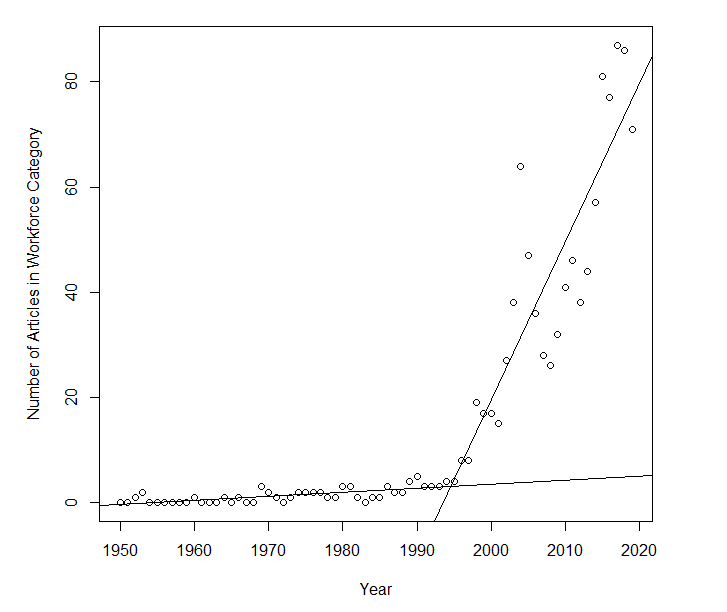

Supplement: S2 Fig — (DOCX) [file pone.0282262.s010.docx]
