# Supplementary material for: An emerging field: An evaluation of biomedical graduate student and postdoctoral education and training research across seven decades
Source: PLoS One. 2023 Jul 25;18(7):e0282262. doi: 10.1371/journal.pone.0282262 (PMC10368290; doi:10.1371/journal.pone.0282262)

S11 Figure: Prediction of publication rates in 2035. An exponential function with the equation below was used to model the data and generate predictions about publication rates over time:

**Number of articles = a*exp(b*(year-1995))**

Parameters a and b were estimated as described in Methods, and are listed in the main article (Table 2), along with predicted publication rates. A graphical representation of the exponential analysis used to make predictions for RA1, RA2, and RA3 is shown below.

###

### RA1


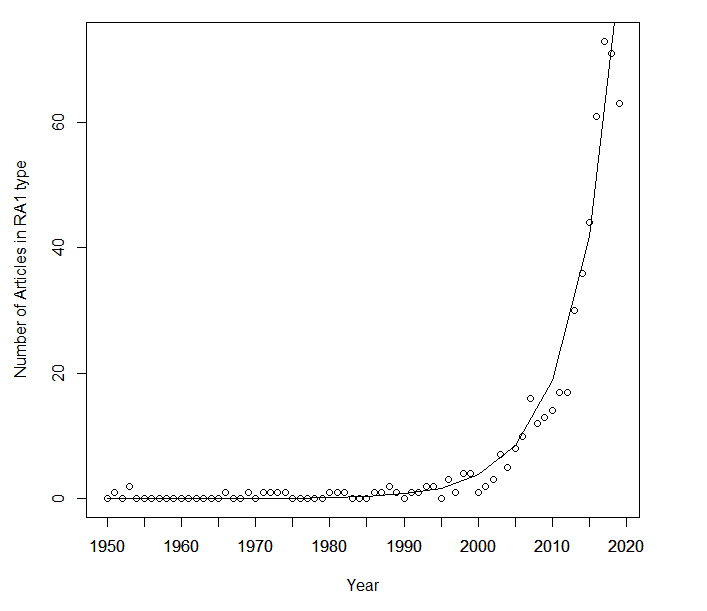


The graph below is the same one as above extended to 2035 using dotted lines.


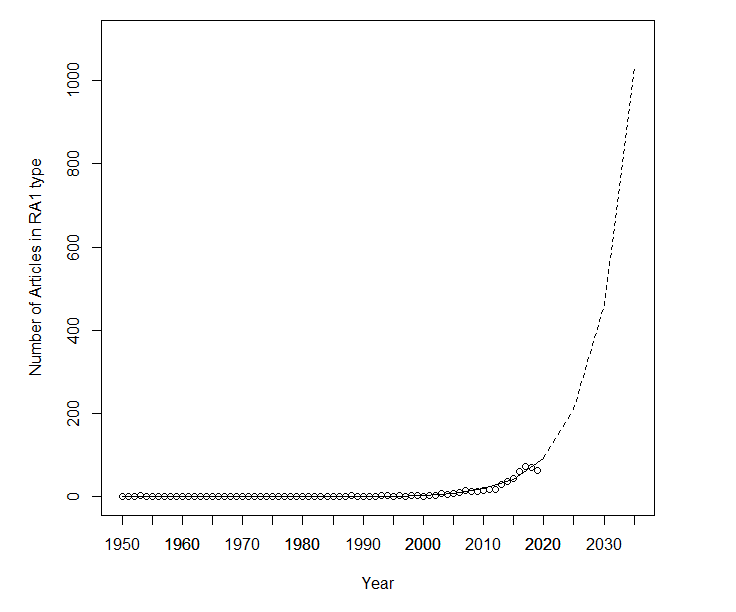


### RA2


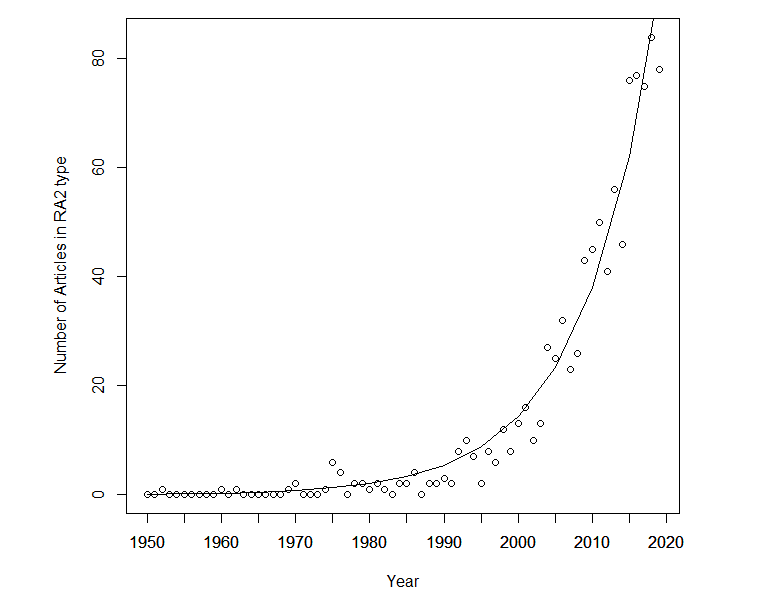


The graph below is the same one as above extended to 2035 using dotted lines.

**
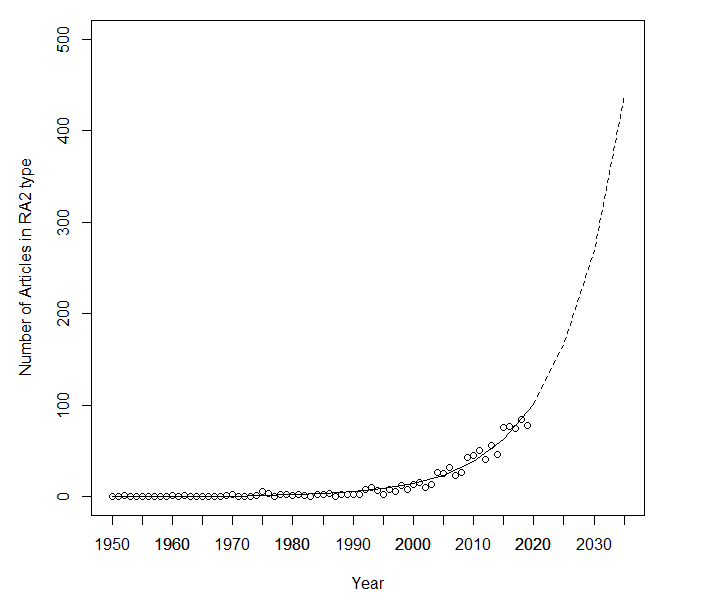
**

### RA3


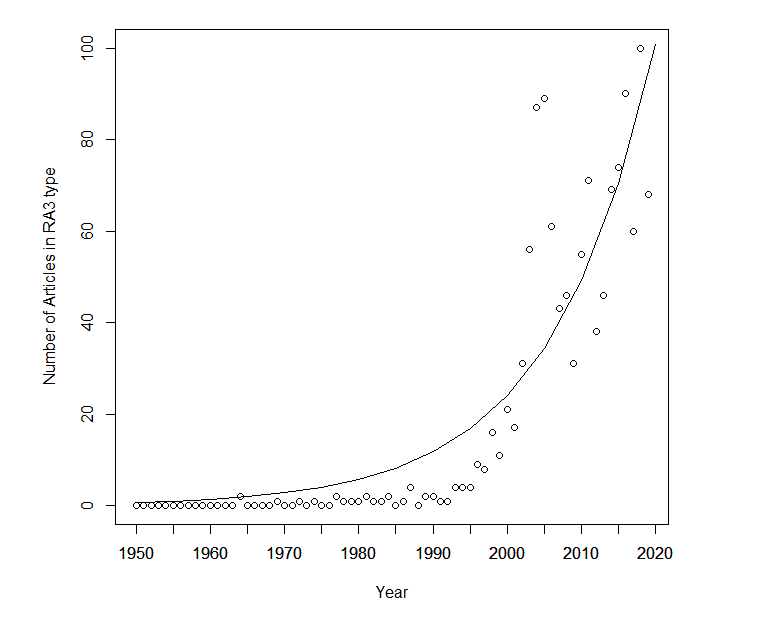


The graph below is the same one as above extended to 2035 using dotted lines.


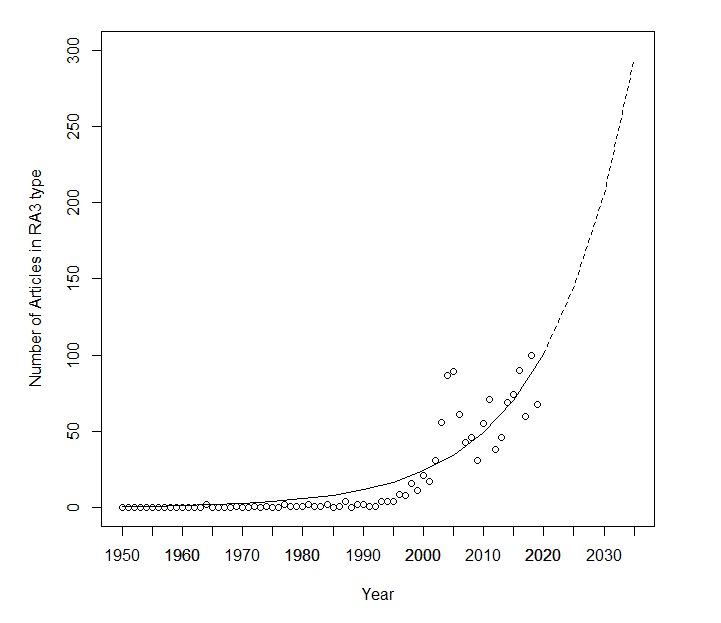

Supplement: S3 Fig — (DOCX) [file pone.0282262.s011.docx]
