# Supplementary material for: An emerging field: An evaluation of biomedical graduate student and postdoctoral education and training research across seven decades
Source: PLoS One. 2023 Jul 25;18(7):e0282262. doi: 10.1371/journal.pone.0282262 (PMC10368290; doi:10.1371/journal.pone.0282262)
Supplement: S1 Data — (DOCX) [file pone.0282262.s012.docx]

S2 Table: Identification of additional search terms (“S2 Table Supplemental Fig Keyword Selection.xlsx”)

Following Search #1, AVW, RL, and JT determined that the search terms were not sufficiently comprehensive, as the results were missing some articles that the authors regularly read and cited. To improve upon the search criteria, AVW, RL, and JT each identified articles that they would expect to be present in a comprehensive literature search on the topic of biomedical graduate and postdoctoral training, for each of the eight thematic subject categories. These articles were fed into the Yale MeSH analyzer to identify additional MeSH terms. In addition, the authors reviewed article titles and abstracts for additional keywords. The “Categories” tab in Excel File S2 contains the preliminary search terms used in Search 1 (Column B), as well as the final search terms used for the study, Search 2 (Columns C – I). The new search terms are colored Red. The remaining tabs contain representative papers that the authors used for identifying the additional search terms.

S8 Table: Full search results with decisions on inclusion and tags (“S8 Table Full_DataSet_2022-04-05.xlsx”)

The spreadsheet contains all 10,913 articles pulled from the search using the defined criteria. The Inclusion/Exclusion decision is listed in column Q. The 2,944 included articles were assigned category tags, listed in column R.

S9 Resource: RIS file bibliography of all articles that met inclusion criteria (“S9 Bibliography_IncludeArticles_2022-04-05_RIS.txt”)

This RIS file is a complete bibliography of all included articles from this study, and can be imported into a variety of citation management applications, including EndNote, Mendeley, and RefWorks. This file should serve as a valuable resource for readers looking to build a library of publications in this field.
